# A systematic review and meta-analysis of the diagnosis and surgical management of carcinoid heart disease

**DOI:** 10.3389/fcvm.2024.1353612

**Published:** 2024-03-20

**Authors:** Jenny Namkoong, Prabha H. Andraweera, Maleesa Pathirana, Dian Munawar, Michael Downie, Suzanne Edwards, Paula Averbuj, Margaret A. Arstall

**Affiliations:** ^1^ Department of Cardiology, Lyell McEwin Hospital, SA Health, Elizabeth Vale, SA, Australia; ^2^ Adelaide Medical School, The University of Adelaide, North Terrace Adelaide, SA, Australia; ^3^ SA Health Library Service, Lyell McEwin Hospital, SA Health, Elizabeth Vale, SA, Australia

**Keywords:** carcinoid heart disease, carcinoid syndrome, meta-analysis, systematic review, endocrinology

## Abstract

**Introduction:**

Carcinoid heart disease (CHD), a complication of carcinoid syndrome (CS), is a rare condition that can lead to right sided valvular heart disease and has been traditionally associated with a poor prognosis. We conducted a systematic review and meta-analysis to explore the accuracy of biomarkers and echocardiography in diagnosing CHD amongst patients who are already known to have neuroendocrine tumours and to assess whether surgical management of CHD leads to a reduction in mortality.

**Methods:**

A systematic literature search of MEDLINE, EMBASE, EBM Reviews, Google Scholar, ClinicalTrials.gov was conducted. All studies on patients with carcinoid heart disease (CHD) reporting on biomarkers, echocardiographic and surgical outcomes were included. The National Heart, Lung, and Blood Institute quality assessment tool was used to assess the methodological study quality. Data analysis was performed using Stata Statistical Software and R Studio, and individual meta-analyses were performed for biomarkers, echocardiographic findings, and surgical outcomes.

**Results:**

A total of 36 articles were included in the systematic review analysis. N terminal pro-brain natriuretic peptide (NTproBNP) and 5-hydroxyindole acetate (5-HIAA) levels were higher in patients with CHD compared with those without CHD. 32% of CS patients had echocardiographic evidence of cardiac involvement, of which 79% involved tricuspid valve abnormalities. Moderate-severe tricuspid regurgitation was the most common echocardiographic abnormality (70% of patients). However, these analyses had substantial heterogeneity due to the high variability of cardiac involvement across studies. Pooled surgical mortality for CHD was 11% at 1 month, 31% at 12 months and 56% at 24 months. When assessing surgical outcomes longitudinally, the one-month surgical results showed a trend towards more recent surgeries having lower mortality rates than those reported in earlier years, however this was not statistically significant.

**Discussion:**

There is not enough data in current literature to determine a clear cut-off value of NTproBNP and 5-HIAA to help diagnose or determine CHD severity. Surgical management of CHD is yet to show significant mortality benefit, and there are no consistent comparisons to medical treatment in current literature.

## 1 Introduction

Carcinoid heart disease (CHD) is an uncommon complication of Carcinoid syndrome (CS), which is a rare syndrome amongst patients with metastatic neuroendocrine tumours (NETs), a neoplasm of enterochromaffin cells that secrete bioactive substances (1). The annual age-adjusted increase in NETs was reported as 6.98 per 100,000 persons in 2012, and the incidence of CS among NET patients has increased from 11% in 2000 to 19% in 2011 (1).

The typical form of CS is characterized by flushing, abdominal cramps, diarrhea and bronchospasm. CS results from an excess secretion of NETs, which can excrete as many as 40 vasoactive products, but predominantly serotonin. It manifests when there is reduced hepatic capacity to metabolize the excess, abnormal secreted vasoactive peptides (2). Rarely, CS may exist in patients without pre-existing liver metastases, such as ovarian and retroperitoneal tumours, where the vasoactive substances enter the systemic circulation via the caval system, bypassing the liver. Atypical CS is rare and mainly occurs in the context of lung NETs, characterized by headache, shortness of breath and extended episodes of flushing. The excess serotonin (amongst other peptides) appears to result in tissue fibrosis in the heart and subsequent CHD (1).

CHD is usually insidious, and most commonly involves the right side of the heart, as the neurohormonal substances break down in the respiratory system before reaching the left heart unless there is a right-to-left shunt such as via a patent foramen ovale (3). Right-sided valves are predominantly involved, leading to right heart failure over time.

As a rare disease, however, there is no clear path for the diagnosis of CHD, which still relies strongly on clinical suspicion. The prognosis of patients with CHD is poor (31% survival rate within 3 years in patients with CHD compared to 69% in those without CHD) (4). There are biomarkers that are well-established to be associated with neuroendocrine tumours and others known to be associated with heart failure, and although it would logically make sense for these biomarkers to be elevated in CHD, they have not been systematically evaluated in literature, nor any cut-off levels that aid in diagnosis been established. Echocardiography as the diagnostic tool for CS, has not been serially analyzed against symptoms, biomarkers and outcomes to be able to guide disease trajectory and management, such as the optimal timing for surgery in CHD. A recent consensus document by European Neuroendocrine Tumor Society (ENETS) has provided a "best practice" proforma recommending information at time of referral to be captured and to create a standardized assessment of patients across sites (5).

There is paucity of data on surgical management of CHD. This may be partly because of the overall paucity of data on CHD, and even less on right heart surgeries for CHD because right-heart surgeries with tricuspid valve replacement or repair and pulmonary valve replacement or repair have been historically conducted mostly in the setting of the patient already undergoing cardiac bypass for an alternative indication. In recent times though, cardiothoracic literature increasingly supports isolated right-sided valvular intervention to improve outcomes (6). Furthermore, it appears that timing of surgical intervention is a significant variable in the degree of mortality benefit for patients undergoing non-carcinoid right-sided valvular surgery (7).

Thus, in view of these lessons in the cardiothoracic field, it is necessary to re-visit its applicability to CHD, which for many years was accepted to have a poor prognosis of 2–4 years mortality from time of diagnosis.

Due to the rarity of CHD amongst an already rare cohort of NETs patients, CHD has been difficult to study. To our knowledge, there are no systematic reviews with meta-analyses assessing the optimal method of diagnosis and management of carcinoid heart disease.

This systematic review explores the question of the accuracy of biomarkers and echocardiography in diagnosing carcinoid heart disease amongst patients who are already known to have neuroendocrine tumours (NETs). It also seeks to answer the question of whether surgical management for carcinoid heart disease will improve mortality.

## 2 Methods

### 2.1 Study design and search strategy

This systematic review and meta-analysis follows the reporting guidelines outlined in the PRISMA statement (Preferred Reporting Items for Systematic Reviews and Meta-Analyses)^1^. The research question informed by PECO is “In patients with carcinoid syndrome, what is the optimal diagnosis method for carcinoid heart disease to minimise mortality and increase quality of life?” The PECO is included in Supplementary Appendix 1.

### 2.2 Eligibility criteria

The review focused on studies that included patients with CHD that reported on biomarkers, echocardiographic findings, and surgical outcomes. Previous reviews of relevant topics and bibliographies of the selected manuscripts were also checked for relevant publications. Only studies published in English in peer-reviewed journals were selected. Case studies, conference abstracts, reviews, editorials, commentaries and book chapters and studies published in languages other than English were excluded.

### 2.3 Search strategy and screening

We conducted a systematic search of literature on MEDLINE, EMBASE, Cochrane Central Register of Controlled Trials, Google Scholar, ClinicalTrials.gov from inception to March 12, 2021 on carcinoid heart disease. An updated search was completed on February 12, 2023. A detailed description of the search strategy is included in Supplementary Appendix 2.

Two reviewers independently screened the titles and abstracts of all studies. Data extraction was also conducted by two reviewers independently (JN and PA). Methodological quality of each study was assessed using the checklist published by the US National Heart, Lung and Blood Institute for case control studies (8). Disagreements between reviewers in title abstract screening, full text screening, data extraction and study quality assessment were resolved by discussion within the team.

### 2.4 Data extraction

Data was extracted from selected studies on study year, design, country, and patient cohort, number of patients (total) and number of patients (CHD) and investigation for measurement.

For two continuous biomarker variables, N terminal pro-brain natriuretic peptide (NTproBNP) level and 5-hydroxyindole acetate (5-HIAA), sample size, mean and standard deviation for both Carcinoid Heart Disease and No Carcinoid Heart Disease groups were included in the analyses. For 5-HIAA, two studies had their values adjusted to be consistent with the ng/l units. A zero standard deviation value was given a value of 0.01.

For 13 dichotomous echocardiographic variables, proportion and 95% confidence interval (CI) of proportion were presented in Forest plots, for each study and then for all the studies combined. For one continuous variable, LVEF in CHD and NoCHD, mean difference and 95% CI were calculated for each study then overall. A variable was included in the meta-analysis if at least 2 of the 16 journal articles involved had sufficient values for that variable (e.g., had a numerator and denominator value). When the numerator was zero it was set to 1 and when the numerator was the same as the denominator it was set to one minus the denominator to avoid leaving out important studies due to extreme proportions not being calculated.

For five dichotomous CHD surgical mortality variables, proportion and 95% CI of proportion are presented in the analysis, for each study and then for all the studies combined. If a study had data for CHD mortality at 1 month, 12 months, 24 months, 36 months or 60 months, it was included in the corresponding forest plot. A number of studies had data for specific time periods, so the years in which surgeries were conducted were included as columns in the Forest plots, with multiple rows in some studies. Meta-regression was performed for each time to death analysis, where the outcome is mortality proportion with associated standard error and the predictor is the year in which the included surgeries started in one model, and the year of the last surgery included in the paper in another model. Mean difference in proportions (i.e., mean difference in proportion of CHD mortality across years), 95% CI and *P* values are obtained from these 10 meta-regressions.

### 2.5 Data analysis

Data analyses were performed using Stata Statistical Software: Release 15.1 College Station, TX: StataCorp LP and R Studio (Version 1.4 1717, 2009–2021). In view of the heterogeneity found for several variables in this meta-analysis, a random-effects model was used throughout. Individual meta-analyses were performed for biomarkers, echocardiographic findings, and surgical outcomes. For biomarkers, the pooled mean difference with its 95% CI was used. For echocardiographic findings values, the pooled weighted proportion was used with its 95% CI. For surgical outcomes, odds ratio (OR) and 95% CI were reported as the outcomes are dichotomous. A *p* value of <0.05 denoted statistical significance. The *I*² statistic was used to evaluate heterogeneity (with *I*² > 50% indicating significant heterogeneity) as was Cochran's Q *P* value (with *p* value < 0.05 indicating significant heterogeneity). A *p* value of <=0.05 denoted statistical significance.

The proportional meta-analyses were done with the Metaprop Stata command to perform meta-analysis of binomial data. In this random-effects model, the observed difference between the proportions and the mean cannot be entirely attributed to sampling error and other factors such as differences in study populations, study designs, etc. could also contribute. Each study estimates a different parameter, and the pooled estimate describes the heterogeneity among the studies and in the case where the variance is zero, this model simply reduces to the fixed-effects model.

A Funnel plot was presented for each variable that had greater than 10 samples to test for publication bias. An Egger's Test was performed for each variable that had greater than 10 samples to test for small study effects.

## 3 Results

**Figure 1:**
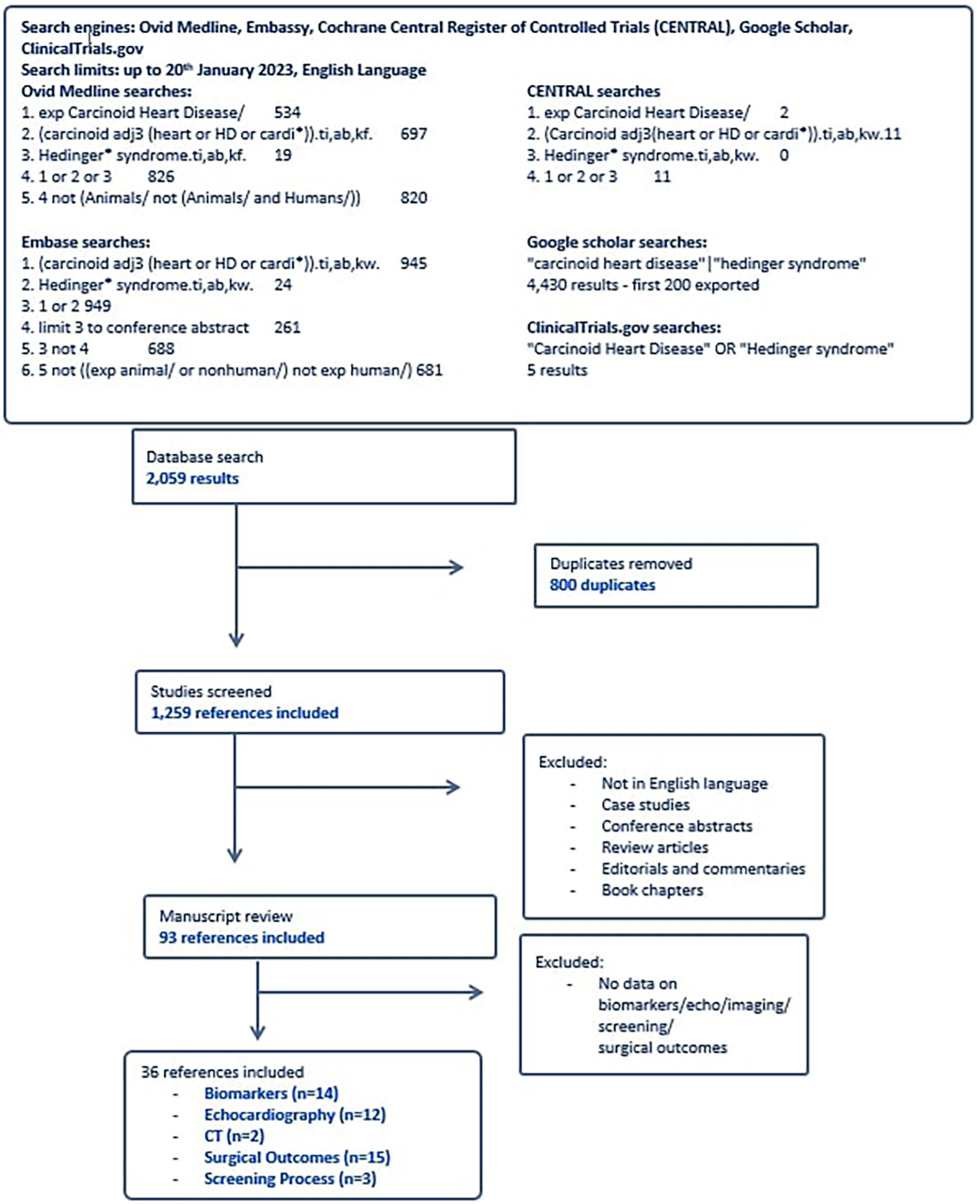
Flow chart of study selection.

A systematic search of the literature from the five databases yielded 2,059 results, of which 800 were duplicates, and the remaining 1,259 were screened by title and abstract (Figure 1). Ninety three articles were selected from the initial screening process by two independent reviewers. Of these, thirty six met criteria for final inclusion in the systematic review analysis. Reasons for excluding certain studies are included in Supplementary Appendix 3. The demographics of each study reported in this systematic review are reported in Table 1. Fourteen studies focused on biomarkers for CHD. Thirteen studies altogether (some from echocardiography-focused papers) had analysable data on biomarkers in CHD, twelve focused on echocardiographic findings, two reported on computerised tomography (CT) findings, and fifteen studies reviewed surgical outcomes with tricuspid valve surgery in the CHD cohort (Table 1). Three studies focused on the screening process for CHD amongst the neuroendocrine metastatic disease population.

**Table 1:** Studies selected from systematic review of literature.

Year	Study	Country	Study design	Patient cohort	Number of patients	Number of CHD patients	Investigation for measurement
Biomarkers							
1989	Lundin et al	Sweden	Prospective, single centre	Metastatic NET	50		ANP
1989	Himelman et al	USA	Prospective, single centre	CS with clinical suspicion of cardiac involvement	30	17	5-HIAA
1998	Denney et al	USA	Prospective, single centre	CS	23	8	5-HIAA
2003	Moller et al	USA	Prospective, single centre	CS	71		5-HIAA
2004	Zuetenhorst et al	Netherlands	Cross section, Single centre	Metastatic NET	32		NT-proBNP, ANP
2008	Bhattacharyya et al	UK	Prospective, single centre	CHD/Metastatic NET	200	39	NT-proBNP
2010	Mansencal et al	France	Prospective, single centre	CS	80		5-HIAA
2011	Haugaa et al	Norway	Cross section, Single centre	CS	89	15	5-HIAA
2011	Komoda et al	Berlin	Cross section, Single centre	CHD with surgery	12	12	5-HIAA
2012	Mokhles et al	Netherlands	Retrospective review	Symptomatic CHD referred for valve surgery	19		5-HIAA
2013	Dobson et al.	UK	Cross section, Single centre	CHD/Metastatic NET	187	37	NT-proBNP, 5-HIAA
2014	Dobson et al	UK	Prospective, 2 centres	CS	100	21	NT-proBNP, 5-HIAA
2018	Alves et al.	Brazil	Retrospective review	CS	42	16	5-HIAA
2020	Baron et al.	France	Prospective single centre	CS	137	49	5-HIAA
2021	Fijalowski et al	Germany	Retrospective, multicenter	CS	107	45	5-HIAA
Echocardiographic parameters							
1989	Himelman et al	USA	Prospective, single centre	CS with clinical suspicion of cardiac involvement	30		TTE at diagnosis & serial
1990	Lundin et al	Sweden	Prospective, single centre	Metastatic NET	31		TTE, TOE
1997	Moyssakis et al	UK	Prospective, single centre	CS	87		TTE at diagnosis
1998	Denney et al	USA	Prospective, single centre	CS	23		TTE at diagnosis & serial
2003	Moller et al	USA	Prospective, single centre	CS	71		TTE at diagnosis & serial
2008	Bhattacharyya et al	UK	Prospective, single centre	CS	150	30	TTE at diagnosis
2010	Bhattacharyya et al	UK	Prospective	CS	252	52	TTE with clinical severity correlation
2010	Mansencal et al	France	Prospective, single centre	CS	80		TTE at diagnosis & serial
2011	Haugaa et al	Norway	Cross section, Single centre	CS	89	15	TTE—Right ventricle
2012	Mokhles et al	Netherlands	Retrospective review	Symptomatic CHD referred for valve surgery	19		TTE pre-operatively
2014	Dobson et al	UK	Prospective, 2 centres	CS	100	21	TTE
2015	Knight et al.	UK	Prospective single centre	CS	30	19	TTE
2019	Nguyen et al	USA	Prospective, single centre	CHD with surgery	240		TTE
2020	Baron et al.	France	Prospective single centre	CS	137	49	TTE at diagnosis & serial
2021	Fijalowski et al	Germany	Retrospective, multicenter	CS	107	45	TTE
2022	Kostianen 2022	Finland	Prospective cross-sectional	CS	65	3	TTE
Surgical outcomes							
1995	Robiolio et al	USA	Retrospective Registry Review	CHD	604	19	Surgical vs. Medical mortality
1995	Connolly et al	USA	Prospective, single centre	Symptomatic CHD	26	26	Surgical vs. Medical mortality
2005	Moller	USA	Retrospective review	CHD	200		Surgical mortality
2008	Castillo et al	USA	Retrospective review	CHD with surgery	10	10	Surgery symptomatic benefit
2011	Komoda et al	Berlin	Cross section, Single centre	CHD with surgery	12	12	Surgical mortality
2011	Bhattacharyya et al	UK	Prospective, single centre	CHDwith surgery	252	22	Surgical mortality & symptoms
2012	Mokhles et al	Netherlands	Retrospective review	CHD with surgery	19		Surgical mortality
2014	Said et al	USA	Retrospective review	Tricuspid valve replacement	64	13	
2015	Connolly et al	USA	Retrospective review	CHD	195		Surgical mortality
2016	Kuntze et al.	Germany	Prospective, single centre	CHD with surgery	39	39	Surgical mortality
2019	Nguyen et al	USA	Prospective, single centre	CHD with surgery	240		Surgical mortality & symptoms
2019	Mortelmans et al	Belgium	Retrospective, single centre	CHD	15	4	Surgical mortality
2020	Yong et al	Australia	Retrospective review	CHD with surgery	20		Surgical mortality
2020	Veen et al	Netherlands	Retrospective review	CHD with tricuspid valve replacement	49		Surgical mortality

### 3.1 Biomarkers

**Figure 2:**
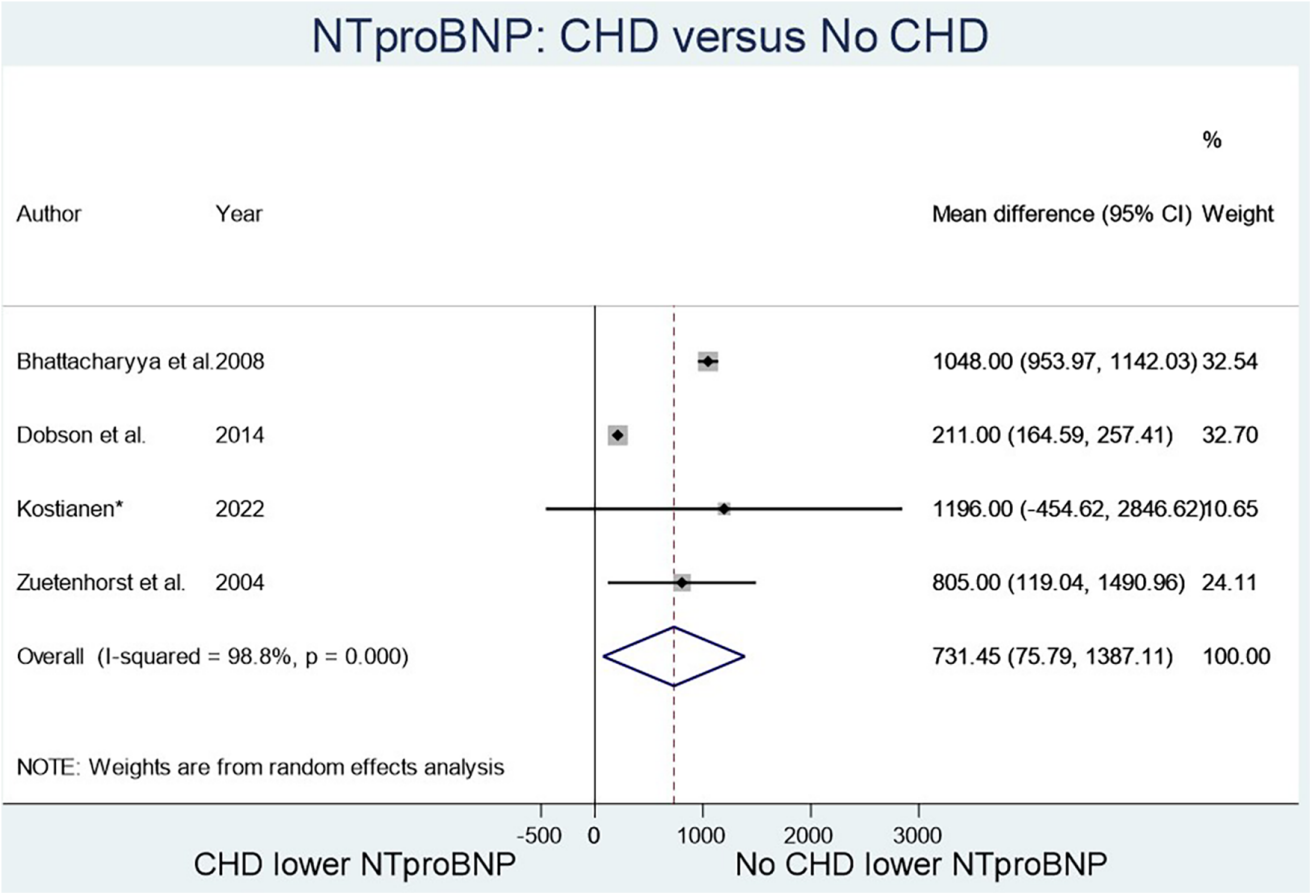
NTproBNP in patients with CHD compared to no CHD.

**Figure 3:**
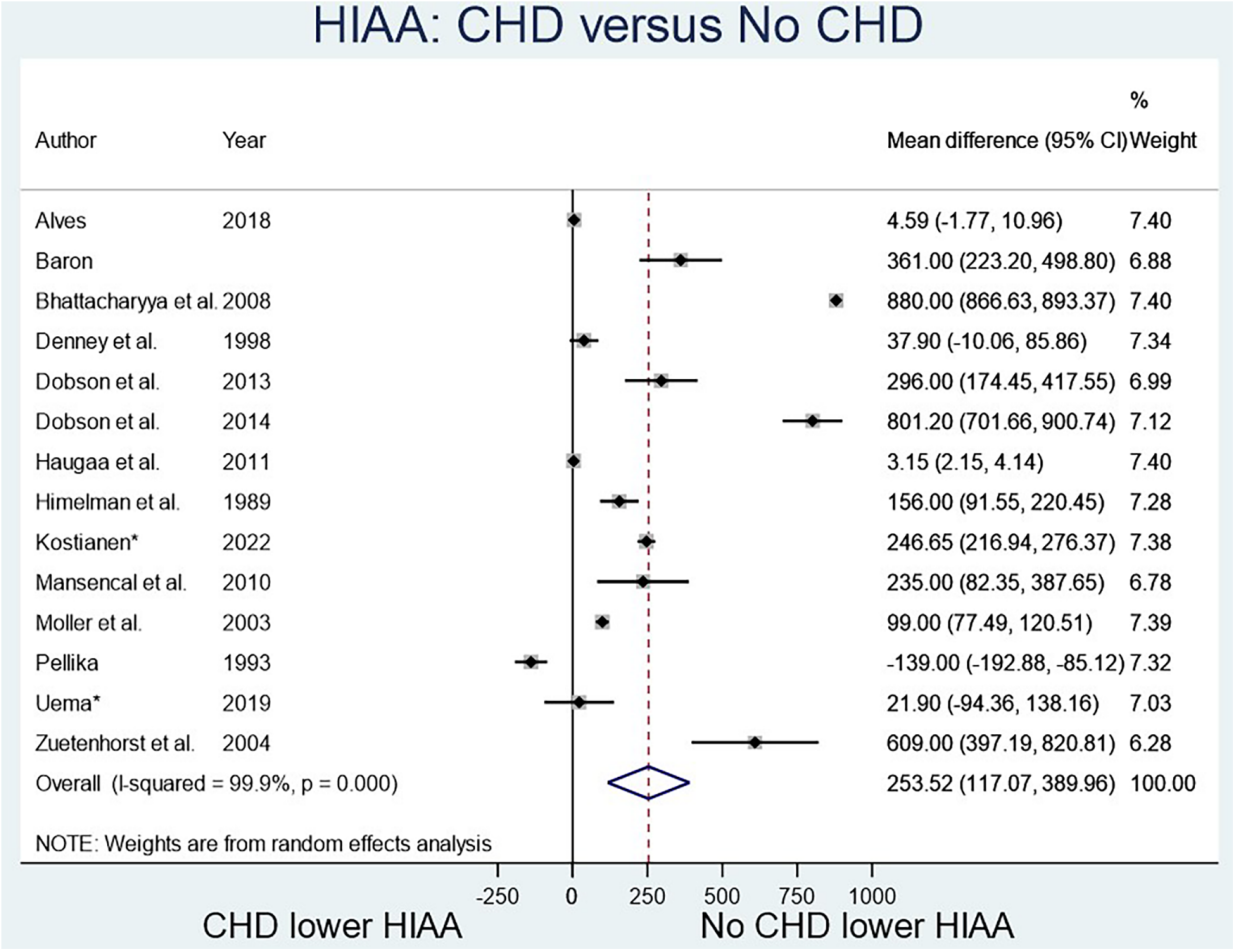
HIAA-5 in patients with CHD compared to no CHD.

The two biomarkers reviewed were NT-proBNP, a marker known to elevate in heart failure, and 5-HIAA, known to elevate with serotonin excretion in CS. Four studies (Figure 2) compared NT-proBNP values in CHD patients vs. those with CS but no CHD (8–12), and fourteen studies (Figure 3) compared 5-HIAA (9, 10, 13–19). NT-proBNP levels were significantly higher in patients with CHD compared with those with no CHD (mean difference (MD) 731.45 (95% CI 75.79–1,387.11, *I*^2^ 98.8%, *p* value < 0.000, Figure 2). Similarly, 5-HIAA levels were significantly elevated in patients with CHD (MD 253.52 (95% CI 111.07–389.96, *I*^2^ 99.9%, *p* value < 0.000 Figure 3A). However, for both meta-analyses, high heterogeneity was observed indicating significant variability between studies.

For NT-proBNP an Egger's test for possible publication bias was not appropriate due to small number (*n* = 4) of studies. For 5-HIAA studies, funnel plot (Figure 3B) shows possible publication bias (10 studies are outside the funnel) and the Egger's Test (*P* value = 0.176) does not show small study effects (Table 2).

**Table 2:** Echocardiographic markers explored in each study.

Study	Cardiac involvement	Tricuspid regurgitation	Tricuspid stenosis	Pulmonary regurgitation	Pulmonary stenosis	Right atria/ right ventricle	Mitral/aortic regurgitation
Himelman et al	✔						
Lundin et al	✔	✔				✔	
Moyssakis et al	✔	✔	✔	✔	✔	✔	
Denney et al	✔						
Moller et al	✔	✔		✔	✔	✔	
Zuetenhorst et al	✔						
Bhattacharyya et al	✔	✔	✔	✔	✔	✔	✔
Mansencal et al	✔						✔
Haugaa et al	✔						
Dobson et al	✔	✔	✔	✔	✔	✔	✔
Baron et al		✔	✔	✔	✔		✔
Kostianen et al. (EF)		✔		✔			
Knight et al. (EF)		✔		✔			

### 3.2 Echocardiography

Of the sixteen studies reporting on echocardiography findings amongst metastatic NET patients to determine extent and type of cardiac involvement (10, 13–15, 18–24), twelve reviewed percentage of cardiac involvement, and eleven papers explored tricuspid valve abnormality with seven papers specifying detection of moderate-severe tricuspid regurgitation (Table 3). Five articles included tricuspid valve thickening, pulmonary regurgitation, six included pulmonary stenosis or right ventricular enlargement as independent echocardiographic markers. Three papers reviewed right atrial enlargement and tricuspid valve retraction as an echocardiographic marker (Table 3).

**Table 3:** Risk of bias within studies from publication and small study bias.

Variable	Egger's test *p* value	Small study bias present	Funnel plot publication bias
Biomarkers			
NT-proBNP	0.562	N/A	N/A
5-HIAA	0.035	*	*
Echocardiogram			
Cardiac involvement	0.048	N/A	N/A
Surgical Outcomes			
1 month	0.000	*	*
12 months	0.649	N/A	N/A
24 months	0.629	N/A	N/A
36 months	0.861	N/A	N/A
60 months	0.602	N/A	N/A

N/A due to less than 10 studies for analysis.

**Figure 4:**
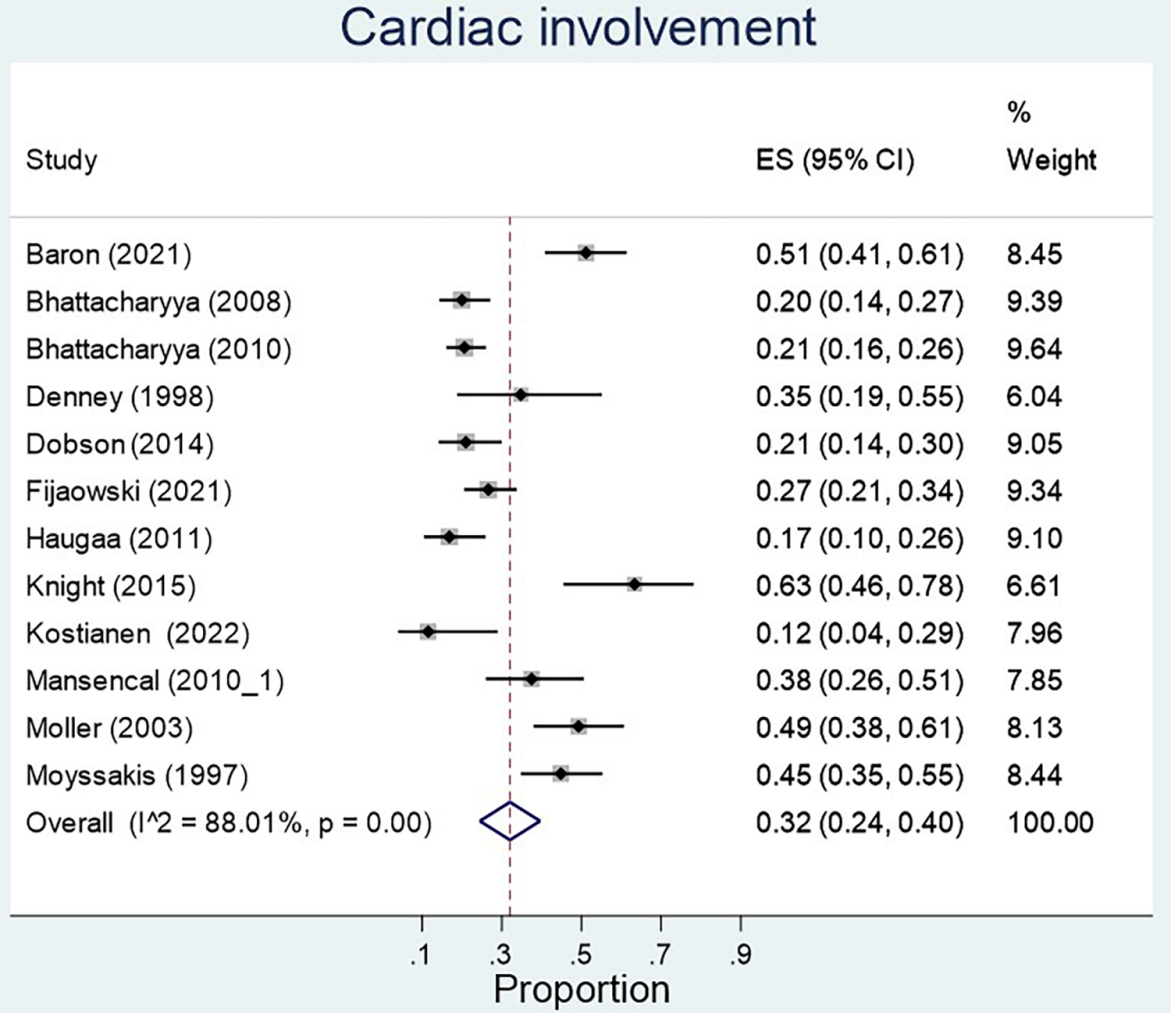
Echocardiographic involvement of cardiac disease in patients with carcinoid syndrome.

**Figure 5:**
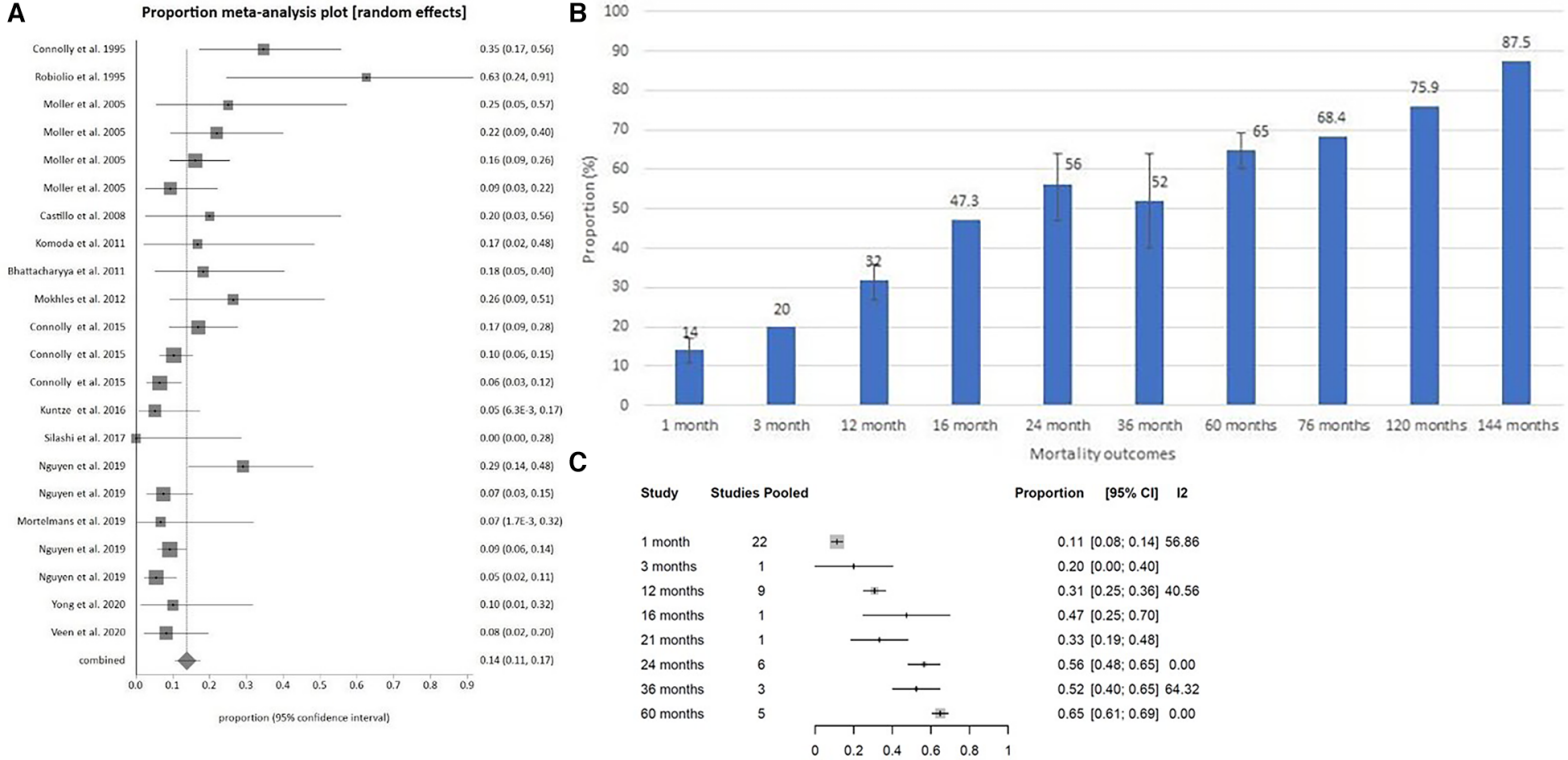
(**A**) Surgical mortality at 1 month for carcinoid heart disease shown chronologically. (**B**) Pooled surgical mortality over time graphically. (**C**) Pooled surgical mortality over time based on meta-regression.

The proportion of carcinoid syndrome patients who had echocardiographic involvement of cardiac disease was 32% across the studies, however heterogeneity across studies was significant (*p* < 0.01) (Figure 4). There was no significant difference in left ventricular ejection fraction between patients with and without cardiac involvement (MD 6.23, 95% CI −7.40–19.86, *I*^2^ 97.7%, *p* value < 0.01, Figure 5). 79% of patients with echocardiographic cardiac involvement had tricuspid valve abnormalities (95% CI 0.69–0.90; *I*^2 ^= 91.96%, *p* < 0.01), of which moderate-severe tricuspid regurgitation was the most common with 70% (95% CI 0.56–0.84; *I*^2^ 81%, *p* < 0.01) in pooled studies (Supplementary Figures S1, S2). Tricuspid valve thickening was documented in 56% (95% CI 0.28–0.84, *I*^2 ^= 95.16%, *p* < 0.01) of the pooled studies, severe tricuspid stenosis in 7% (95% CI 0.01–0.13 *I*^2^ = 69.6%, *p* = 0.01), and mild tricuspid regurgitation in 19% (95% CI 0.00–0.38; *I*^2^ = 85.17%, *p* < 0.01). However all analyses reported significant heterogeneity (Supplementary Figures S3–S5).

Significant pulmonary regurgitation was documented in 21% (95% CI 0.06–0.36; *I*^2^ = 81.04%, *p* < 0.01) of the pooled CS study cohort and mild pulmonary regurgitation in 40% (95% CI 0.29–0.50 = *I*^2^ 34.40%, *p* = 0.19), whilst pulmonary stenosis was noted in 43% (95% CI 0.24- 0.63; *I*^2^ = 90.24%, *p* < 0.01) (Supplementary Figures S6–S8). Significant mitral regurgitation was less common, being documented in 11% (95% CI 0.05–0.17; *I*^2^ = 55.55%, *p* = 0.05) as was aortic regurgitation documented in 10% (95% CI 0.06–0.14; *I*^2 ^= 22.34%, *p* < 0.01) of the pooled echocardiographic analysis, with low-moderate heterogeneity reported for both analyses (Supplementary Figures S9, 10). The frequency of left-sided valve lesions was more consistent in the studies with results falling within the 95% confidence interval funnel plot, compared with the results for the frequency of right-sided valve lesion echocardiographic findings (Supplementary Figure S11).

In regard to cardiac chambers, right atrial enlargement abnormalities was commonly found (74%) but this wasn’t statistically significant (95% CI 0.45–1.03), whilst right ventricular enlargement occurring in 43% of echocardiographic pooled findings was less common (95% CI 0.23–0.63). For all of these analyses, the heterogeneity for the studies was significant at *I*^2 ^> 85% for all analyses (Supplementary Figures S12, S13).

### 3.3 Surgical outcomes

The fifteen studies exploring mortality outcomes from surgery for CHD included studies published from 1995 to 2020 (16, 18, 24–36), representing 766 surgeries from the year 1981 to 2017 (Table 1). Most of these were tricuspid valve replacement surgeries, although the pooled cohort did include tricuspid valve repairs and multi-valve surgeries with tricuspid and pulmonary valve replacement. Exact surgical techniques were not specified, and in many studies the mortality analysis did not separate isolated tricuspid valve replacements from other CHD surgeries. The detail on number of valve replacements across studies where recorded is highlighted in more detail in Table 4.

**Table 4:** Surgical indications for participants in all studies.

Study	*N* = Tricuspid valve replacement (single or multi-valve)	*N* = TVR single valve surgery	*N* = Double-valve (TVR + 1 additional valve)*	*N* = Triple valve (TVR + 2 additional valve)*	*N* = Quadruple valve((TVR + 3 additional valve)*
Robiolio et al.	8	6	2	-	-
Connolly et al.	26	16	5	4	1
Moller et al.*	87	52	NS	NS	NS
Castillo et al.	11	-	10	1	-
Komoda et al.	9	6	-	-	-
Bhattacharyya et al.	22	3	15	2	2
Mokhles et al.	19	4	15	-	-
Said et al	64	64	-	-	-
Connolly et al. 2015**	195	NS	NS	NS	NS
Kuntze et al.	39	34	5	-	-
Nguyen et al.	237	32	175	24	9
Mortelmans et al.	15	1	12	-	2
Yong et al.	19	1	18	-	-
Veen et al.^a^	98	27	NS	NS	NS

^a^ Concomitant surgery (TVR + PVR *n* = 26, + AVR *n* = 16, + MVR *n* = 36).

* >35 patients had surgery for at least 2 valves.

** Pulmonary, mitral or aortic valve disease warranting intervention was detected in 147, 21 and 18 patients respectively.

**Figure 6:**
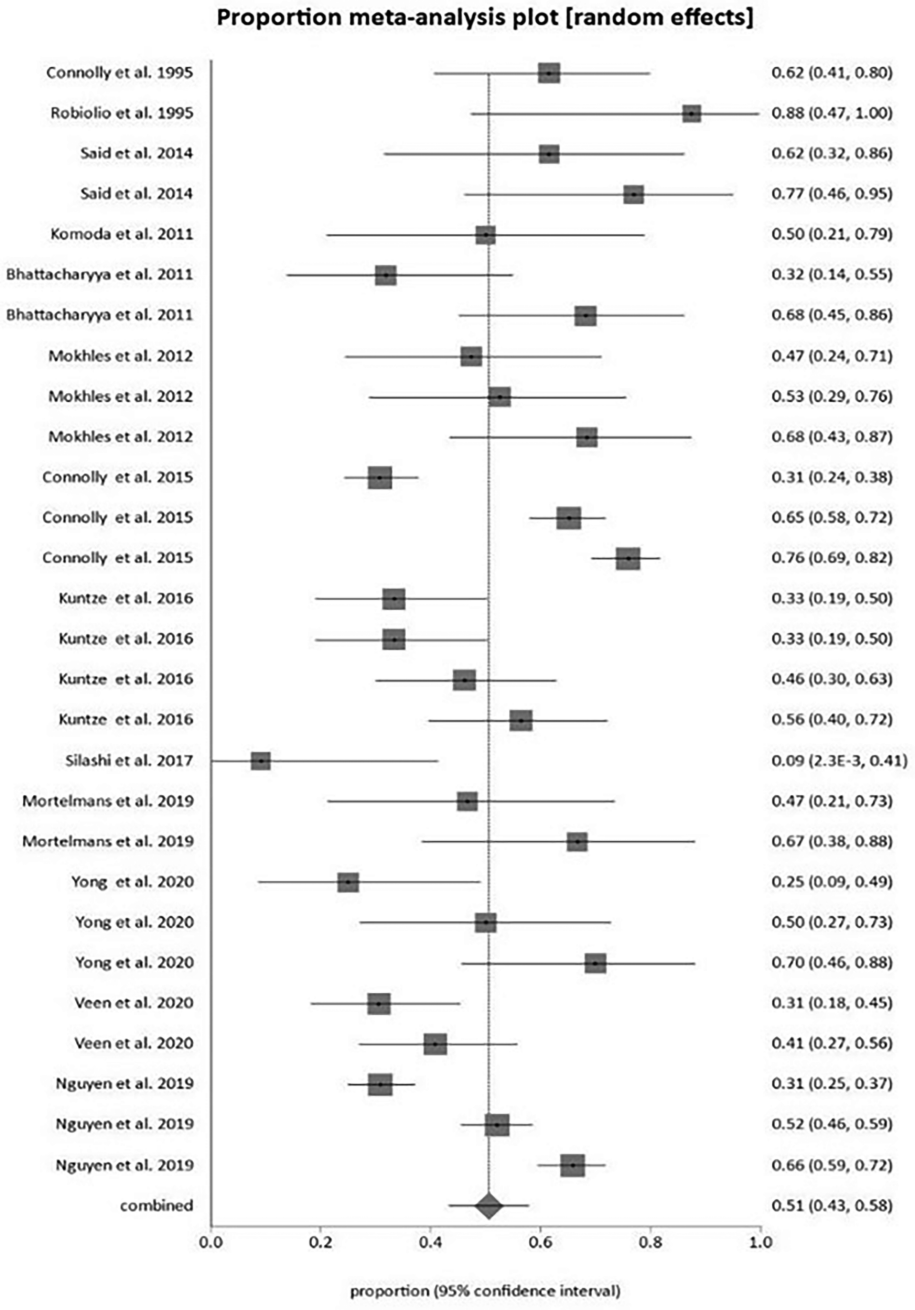
Surgical mortality at 12 months carcinoid heart disease shown chronologically.

Pooled surgical mortality for CHD was 12% at 1 month, 31% at 12 months and 56% at 24 months, 52% at 36 months, and 65% at 60 months (Supplementary Figures S14–S18). The heterogeneity of these analyses were low to moderate: *I*^2 ^= 54.37%, 40.56% and 0% respectively). When looking at surgical outcomes over time, the one-month surgical outcome results showed a modest trend towards more recent surgeries having lower mortality rates than earlier surgeries (Figures 5A–C). At 12 months, the surgical outcome mortality did not show a chronological trend toward improvement over time (Figure 6).

### 3.4 Tables

The risk of bias assessment in the included studies is shown in Table 2 and the supplementary material (Supplementary Table S1). In the surgical outcome statistical analysis for one month mortality, small study bias and publication bias were present (37). In the 5-HIAA outcome, small study bias and publication bias were present (Figure 3B). Although it appeared as though the risk of bias reduced for surgical outcomes with time, at 12 months, 24 months and 36 months compared with the 1 month outcomes, this is not possible to confidently conclude as funnel plots and Egger's test to assess for possible publication bias are not applied to forest plots with less than ten studies as in this case, as when there are fewer studies the power of the tests is too low to distinguish chance from real asymmetry (37).

For the same reason, small study bias and publication bias were not able to be assessed for NTproBNP in CHD, nor for each echocardiographic variable in CHD.

## 4 Discussion

### 4.1 Summary of findings

This systematic review adds to the current evidence in showing that elevated biomarkers for CS (5-HIAA) and heart failure (NT-proBNP) are further elevated in CHD. However, it reveals that the presentation of CHD, reflected by the heterogeneity across studies and poor quality of some studies, is so varied that it is difficult to determine a cut-off value for diagnosis of CHD for either of the biomarkers.

This review has established the frequency of specific echocardiographic findings in the context of CHD amongst a CS and metastatic NET population. However, these findings are highly heterogeneous across studies, therefore, we could not infer specific criteria for echocardiographic findings for CHD based on the literature collectively in the format of a meta-analysis.

The most notable and unique finding of this systematic review is tracking the mortality of surgical management of the tricuspid valve for CHD chronologically. This revealed that although there is a non-statistically significant trend towards improved surgical outcomes over time, there is not yet any clear evidence that surgical management offers lower mortality today than it did in previous decades. There are also no studies directly comparing surgical management with medical management with respect to mortality or morbidity.

The exploration of the review looking at biomarkers in CHD was limited by the few available studies, as our inclusion criteria reviewed only articles with biomarker values in a CHD population. Our analysis showed that there was a correlation between higher NT-proBNP and 5-HIAA in CHD compared to those without CHD. Although a trend of elevation of levels in the presence of CHD could be seen with both biomarkers, the data is too varied and heterogeneous to determine a cut-off value for diagnosis of cardiac involvement amongst CS or metastatic NET patients. One study in the review by Bhattacharyya suggested a cut-off value of NT-proBNP 260 pg/mL to use to further investigate those metastatic NET patients with an echocardiogram (8), which is what has been similarly recommended in the recent clinical guidelines by the ENETS committee (a cut-off level of 235–260 pg/ml). NT-proBNP is considered the most sensitive marker for presence and severity of CHD and should be measured in all patients with high u5-HIAA even without CS (5). The clinical guidelines also specified that a Urinary-HIAA secretion >=50 umol is compatible with the diagnosis of CS and is recommended in screening of all patients. The ENETs guideline highlights that while U-5HIAA and NT-pro-BNP are good clinical markers for CS, prognostic markers of aggressive CS and CHD are still required, particularly ones that encompass the broad symptomology of CS. Our review supports the need for more predictive markers given the variability of population data across studies for the current biomarkers.

In reviewing the echocardiographic findings in CHD, it became clear that given CHD is largely diagnosed from echocardiography and only rarely confirmed from histological analysis with biopsy or surgery. Recently, Hofland et al. (2021) have devised a Synoptic Reporting of Echocardiography in CHD which derives a total carcinoid heart disease score based on TTE examination, which may be useful for standardizing care and follow-up of patients with CHD, including referral for surgery. Our findings are in line with Hofland and colleagues, reporting based on meta-analysis that the tricuspid valve is the most affected in CHD (79%), however the report specified 90% based on a large study by Bachhyra et al. The authors acknowledged the lack of standardized TTE reporting as a major challenge in this setting. Based on our review findings, we also recommend standardized timing of referral for NETs patients for echocardiography and cardiology review.

In terms of the surgical literature on CHD, two issues emerged. Firstly, when the study included more than thirty cases, the different types of surgical procedures (tricuspid repair vs. replacement vs. multi-valvular replacement with both tricuspid and pulmonary valves) were not separated in the analysis, therefore affecting the rate of mortality as severity of surgery and operative risk will vary across studies. Secondly, the only consistent objective outcome for comparison was mortality. Although the timeframe at which mortality was measured was not consistent across the studies, most studies gave a 30-day mortality outcome, then varied in providing 12-month and up to 144-month data. The trend that 30-day mortality has declined over time is in line with what is reported in ENETS clinical guidelines, and surgical valve replacement is still considered the best practice for managing CHD, however we have not shown a statistically significant improvement over time. Future studies should compare surgical and non-surgical interventions for CHD. The ENETS guidelines do recommend that prognostic indicators of CHD are needed to aid in accurate timing for surgical intervention.

### 4.2 Limitations

This systematic review had major limitations. The first and foremost, majority of the analyses were heterogeneous due to the variability in population data and high variability of cardiac involvement. CS is a multifactorial condition in which a broad range of symptoms, physical manifestations and biochemical findings need to be considered in the diagnosis. The 2022 ENETS guidance paper for CS and CHD provides a comprehensive guide for diagnosis of CS which can be referred to. Another limitation is that our analysis on surgical mortality does not take into account the change in this approach. However we believe that the chronological assessment of reduced mortality over time indicates an improvement in surgical management overall. Third, we were not able to adjust for other complexities of CS including the status of the tumor, CS status, liver synthetic function, presence of right heart failure and nutritional status. While our findings are based on studies of high heterogeneity, it is important to acknowledge that the findings of this systematic review are in line with what is reported in recent guidelines, however specific cut-off values cannot be determined without future studies being uniform in their diagnosis and assessment of CS.

The limitation in the literature has been a lack of trials, RCTs or otherwise, comparing medical to surgical management in the same population group. An RCT for such a rare condition is understandably difficult, and in the absence of RCTs the next best option would be to pool studies of surgically managed patients to compare with pooled studies of medically treated patients, aiming to control for variables including year of diagnosis and year of surgery to account for improvements in both medical and surgical treatments over time. However, this strategy will still not be able to account for the inherent selection bias of those who were offered surgery and those who were not; on one hand the surgical cohort possibly being more comorbid with worse prognosis to be offered surgery as a last-resort option, whilst on the other hand they may have to have been of better baseline functional state to be clinically deemed as able to survive a major operation. The authors of this systematic review did not conduct such an analysis as such selection biases would be impossible to discern during the initial search strategy.

### 4.3 Conclusion

CHD is an important but rare condition with no published RCTs of diagnosis or management performed. Whilst noted to be involved in the presence of CHD, neither the biomarkers NTproBNP and 5-HIAA, nor a variety of echocardiographic findings, could be validated as a clinical metric to diagnose CHD or assess its severity. Surgical interventions studies for CHD showed a reduction in mortality over time but there were no consistent comparisons to medical treatment. Although the recent ENETS guideline attempt to improve the clinical diagnosis and management of CHD, the data available to inform these guidelines is weak as demonstrated by our meta-analysis. Large international registries and carefully designed clinical trials for small cohorts are needed to better understand the expected clinical markers at each stage of CHD progression, with correlation with morbidity and mortality to determine the optimal management of this condition with a generally poor prognosis when left untreated.

## Data Availability

The original contributions presented in the study are included in the article/**Supplementary Material**, further inquiries can be directed to the corresponding author.
