# Supplementary material for: A systematic review and meta-analysis of the diagnosis and surgical management of carcinoid heart disease
Source: Front Cardiovasc Med. 2024 Mar 20;11:1353612. doi: 10.3389/fcvm.2024.1353612 (PMC10987853; doi:10.3389/fcvm.2024.1353612)
Supplement: Supplementary file 1 [file Table1.docx]

Supplementary Material


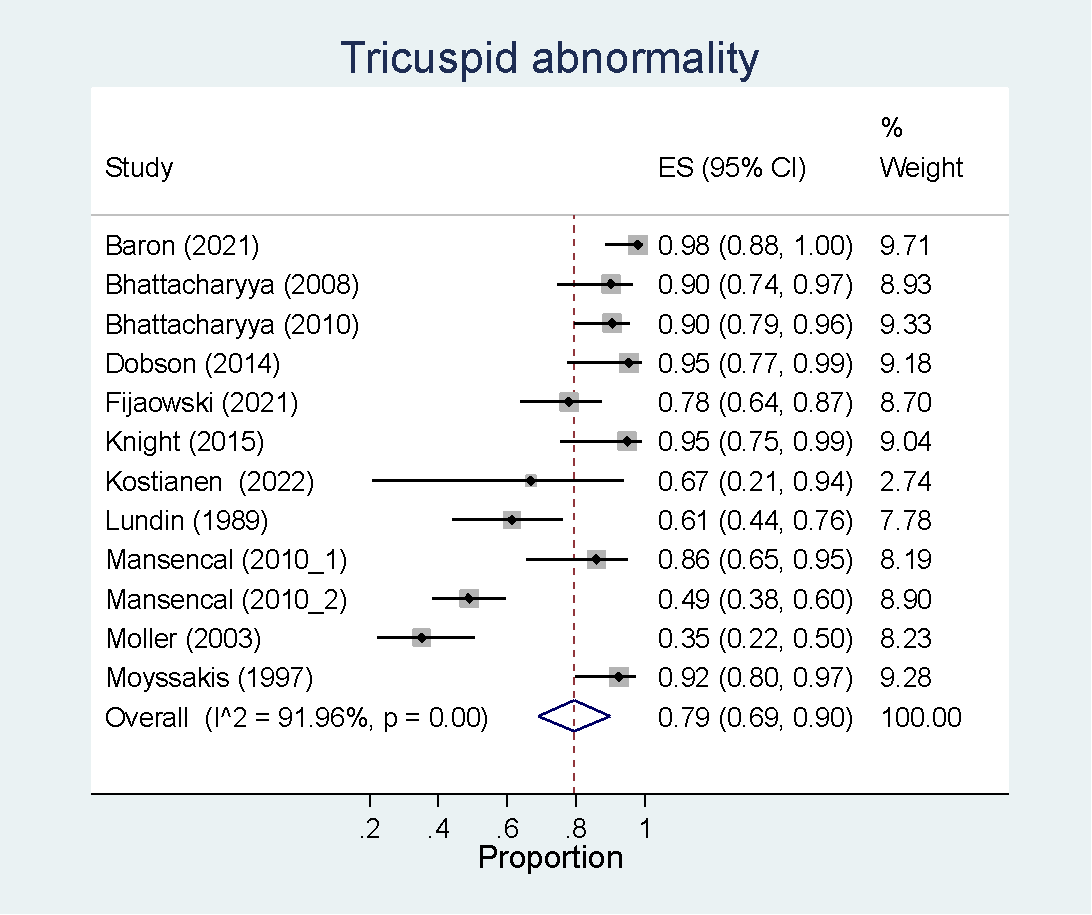


**Supplementary Figure 1.** ***Echocardiographic finding of tricuspid abnormality in CS patients***


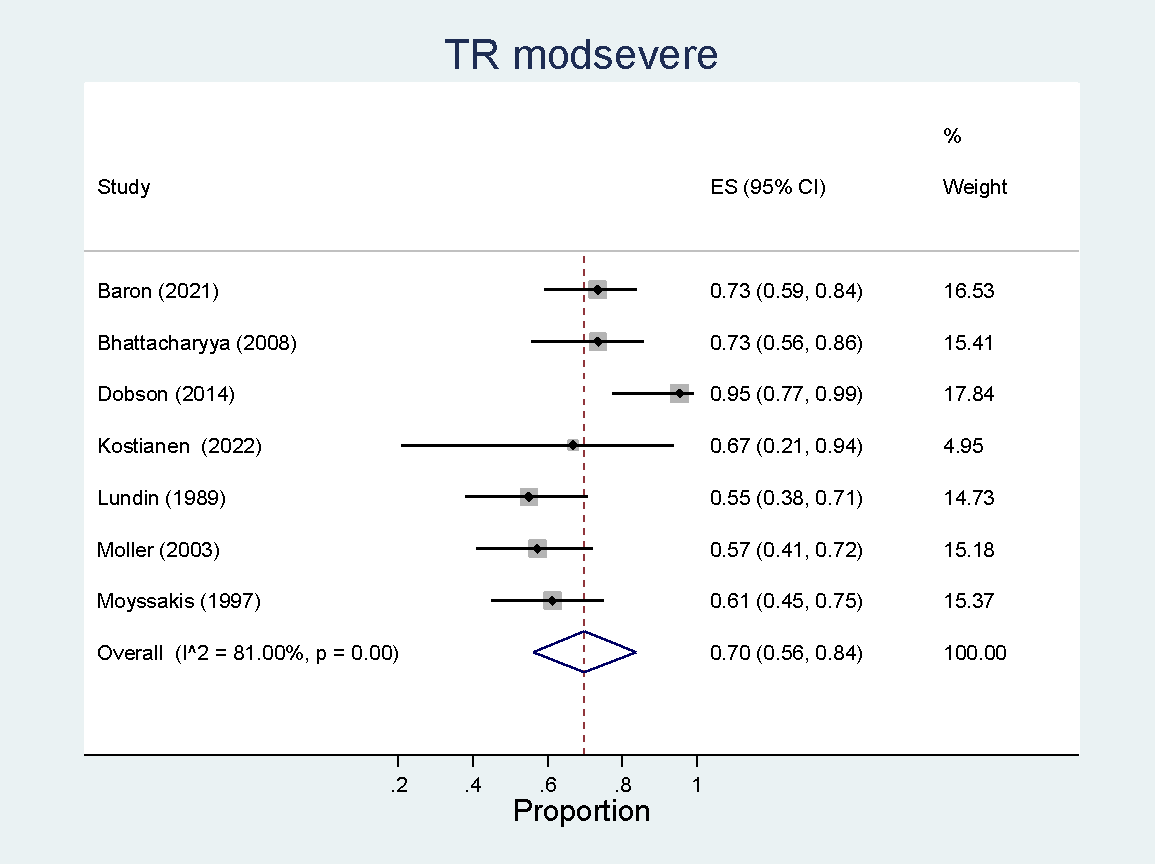


**Supplementary Figure 2.** ***Echocardiographic finding of moderate-severe tricuspid regurgitation (TR) in CS patients***


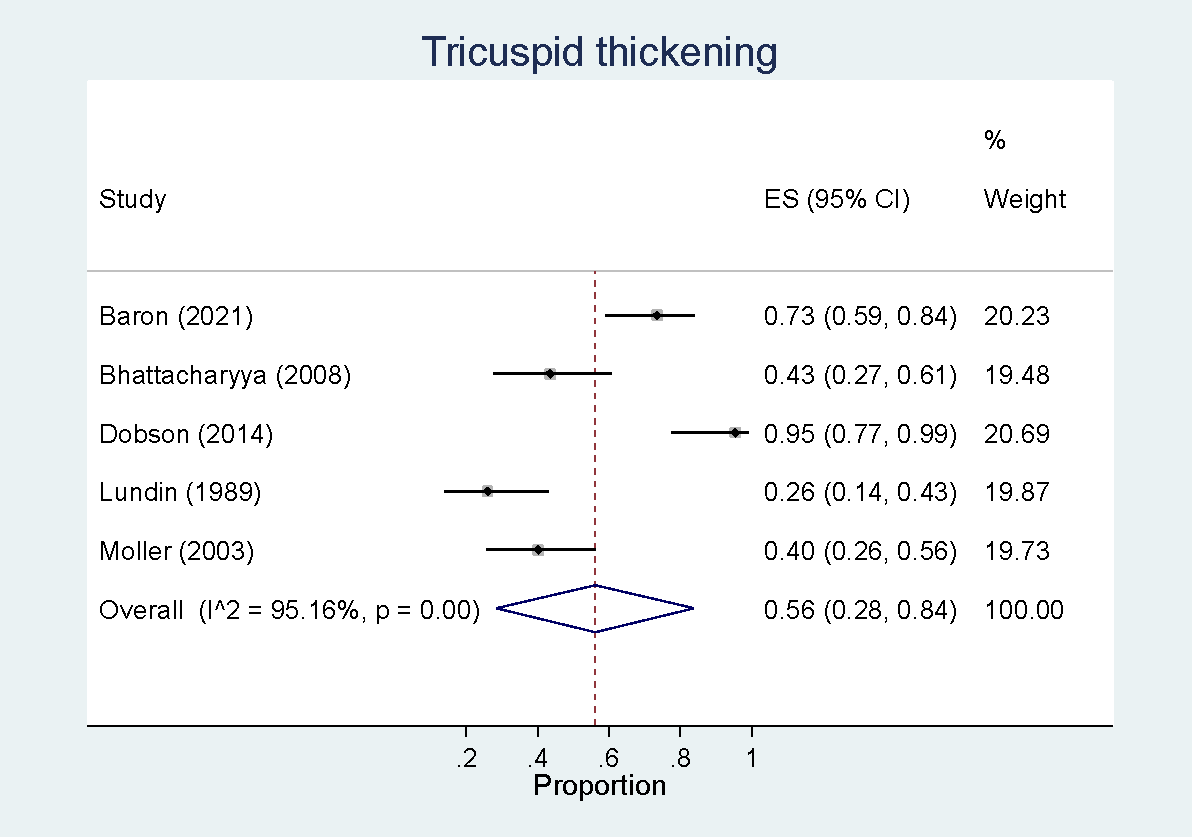


**Supplementary Figure 3.** ***Echocardiographic finding of tricuspid valve thickening in CS patients***


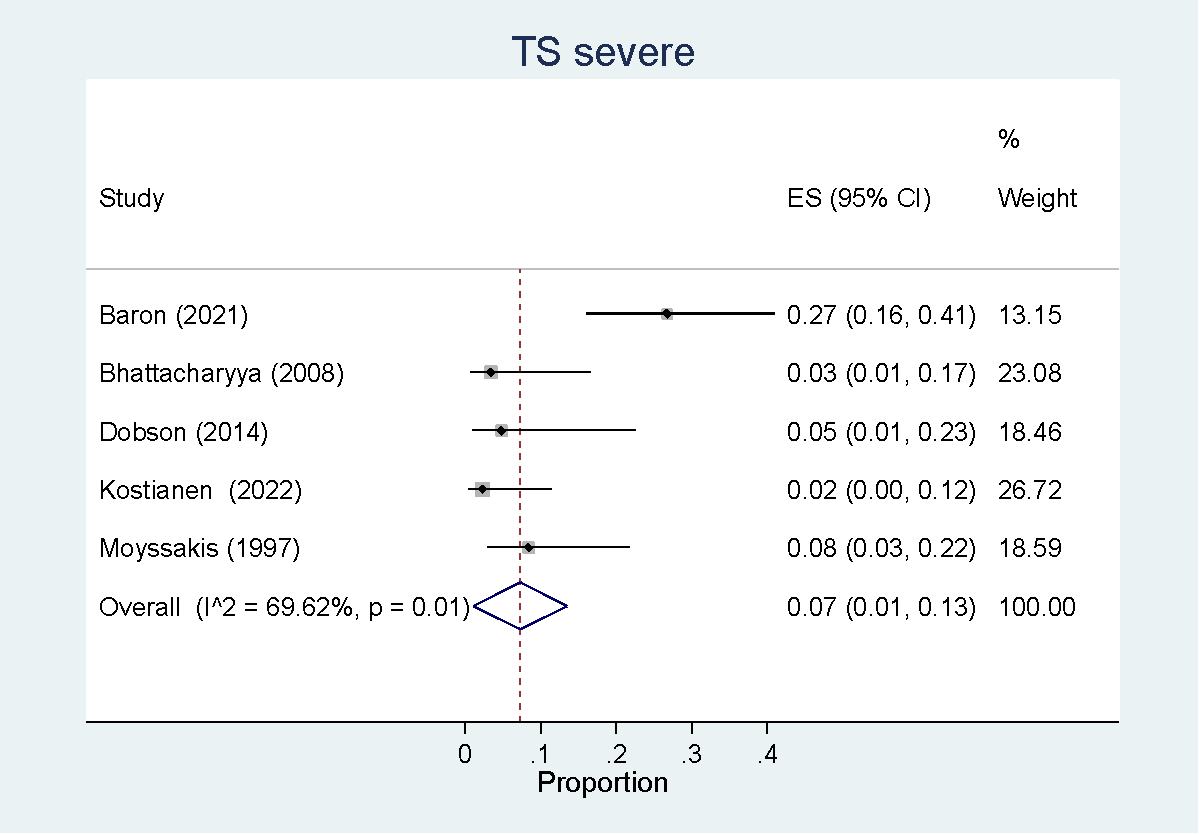


**Supplementary Figure 4.** ***Echocardiographic finding of severe tricuspid stenosis (TS) in CS patients***


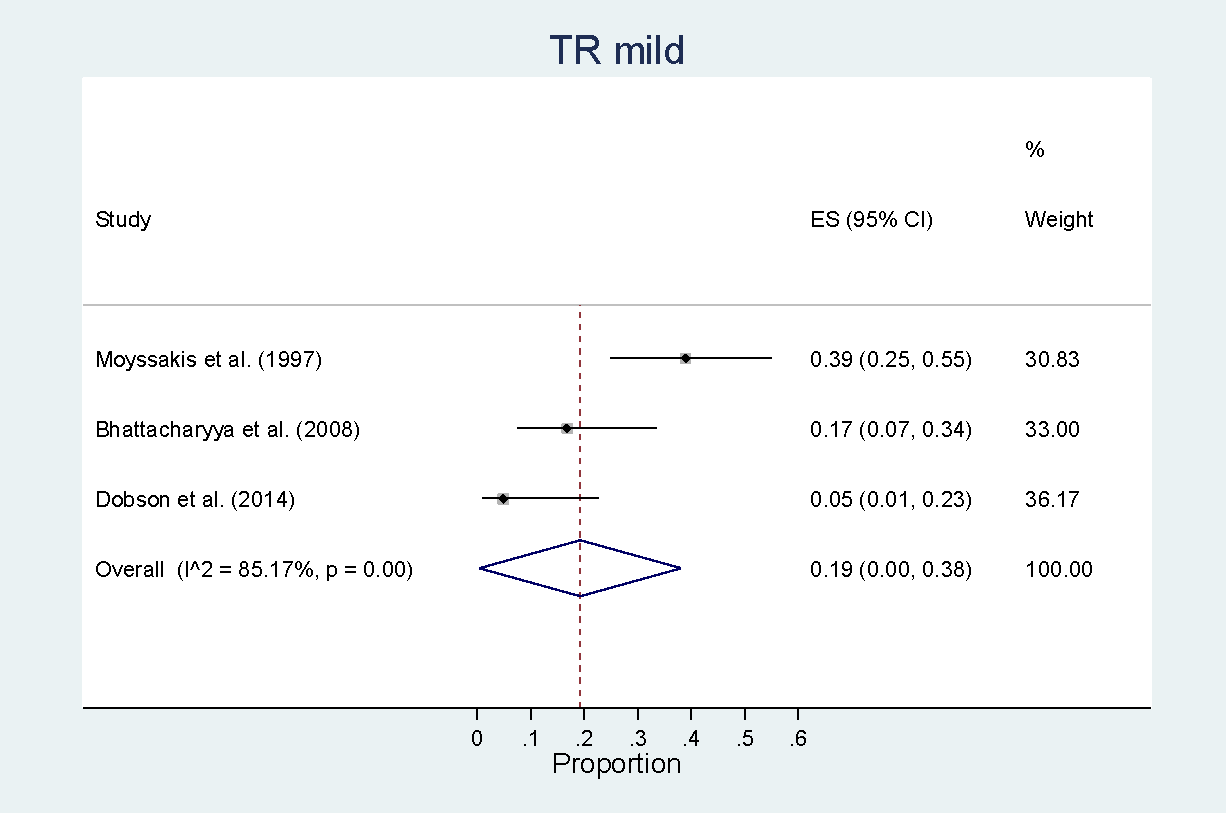


**Supplementary Figure 5.** ***Echocardiographic finding of mild tricuspid regurgitation (TR) in CS patients***


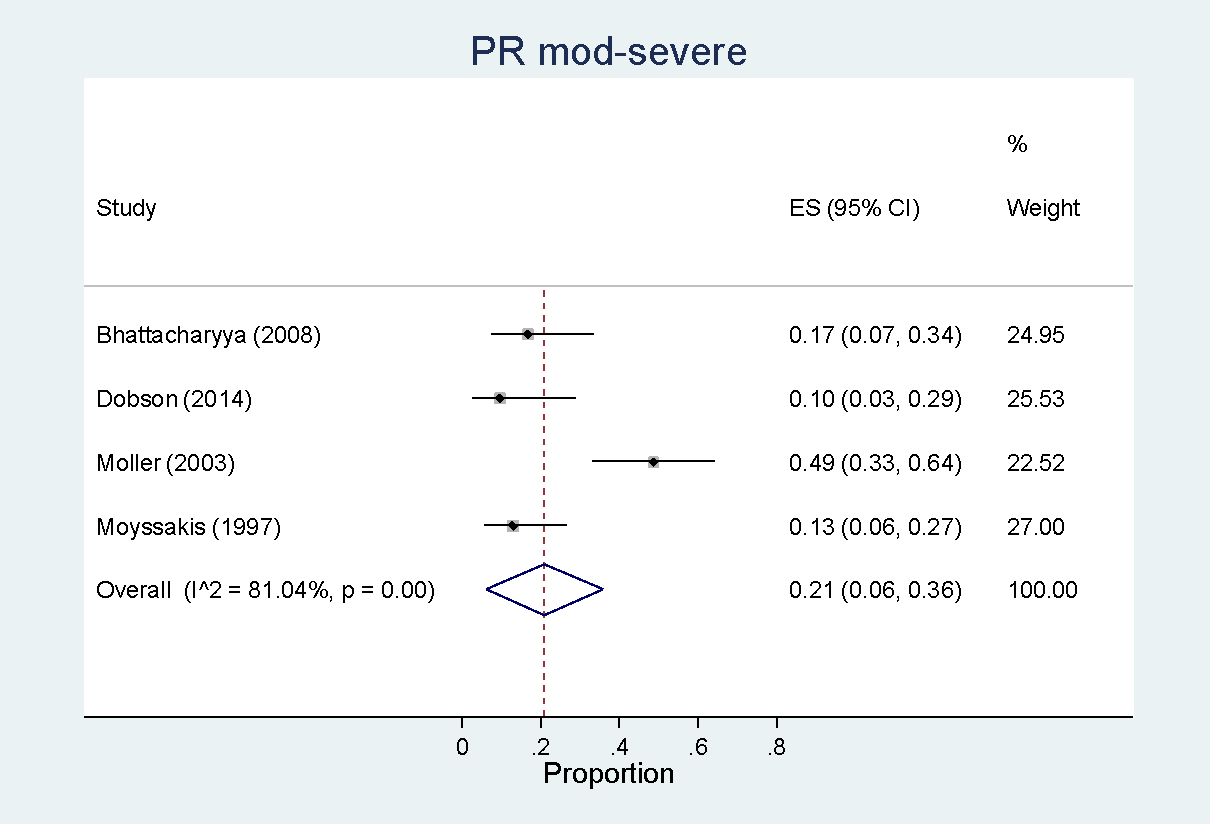


**Supplementary Figure 6.** ***Echocardiographic finding of moderate to severe pulmonary regurgitation (PR) in CS patients***


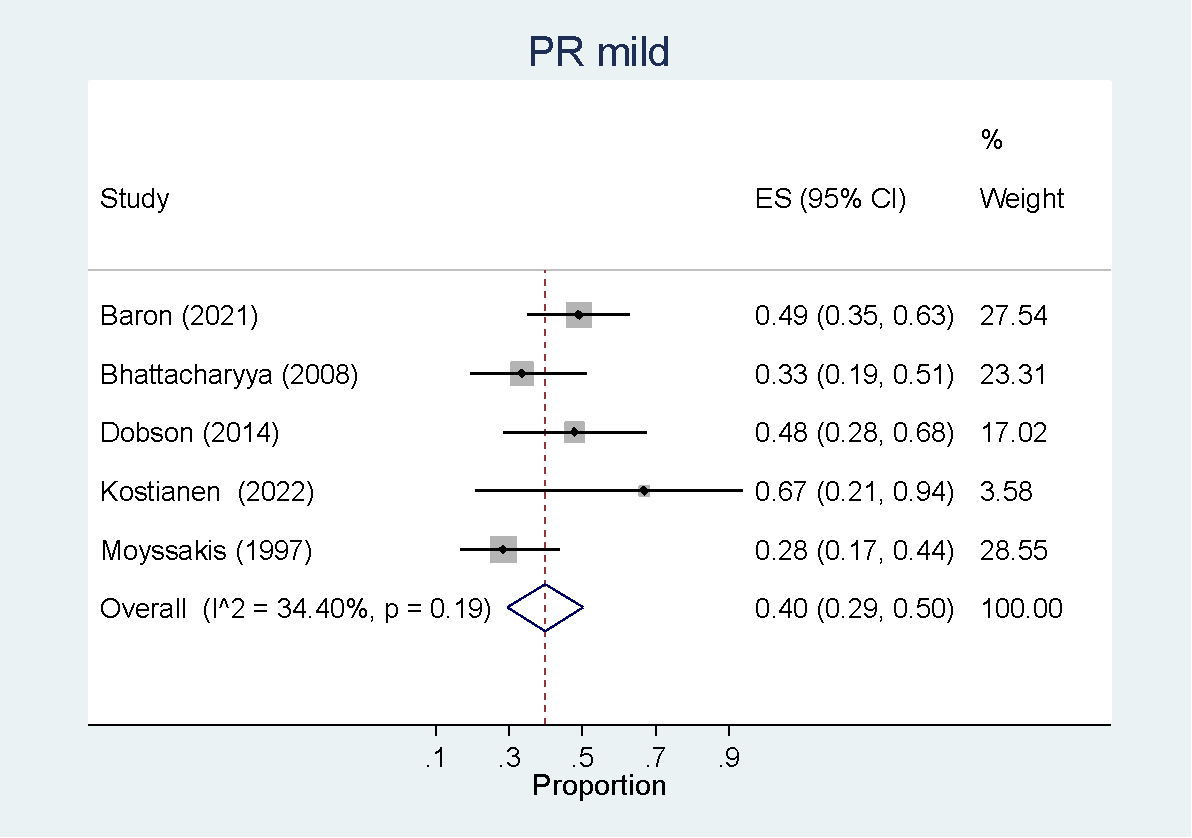


**Supplementary Figure 7.** ***Echocardiographic finding of mild pulmonary regurgitation (PR) in CS patients***


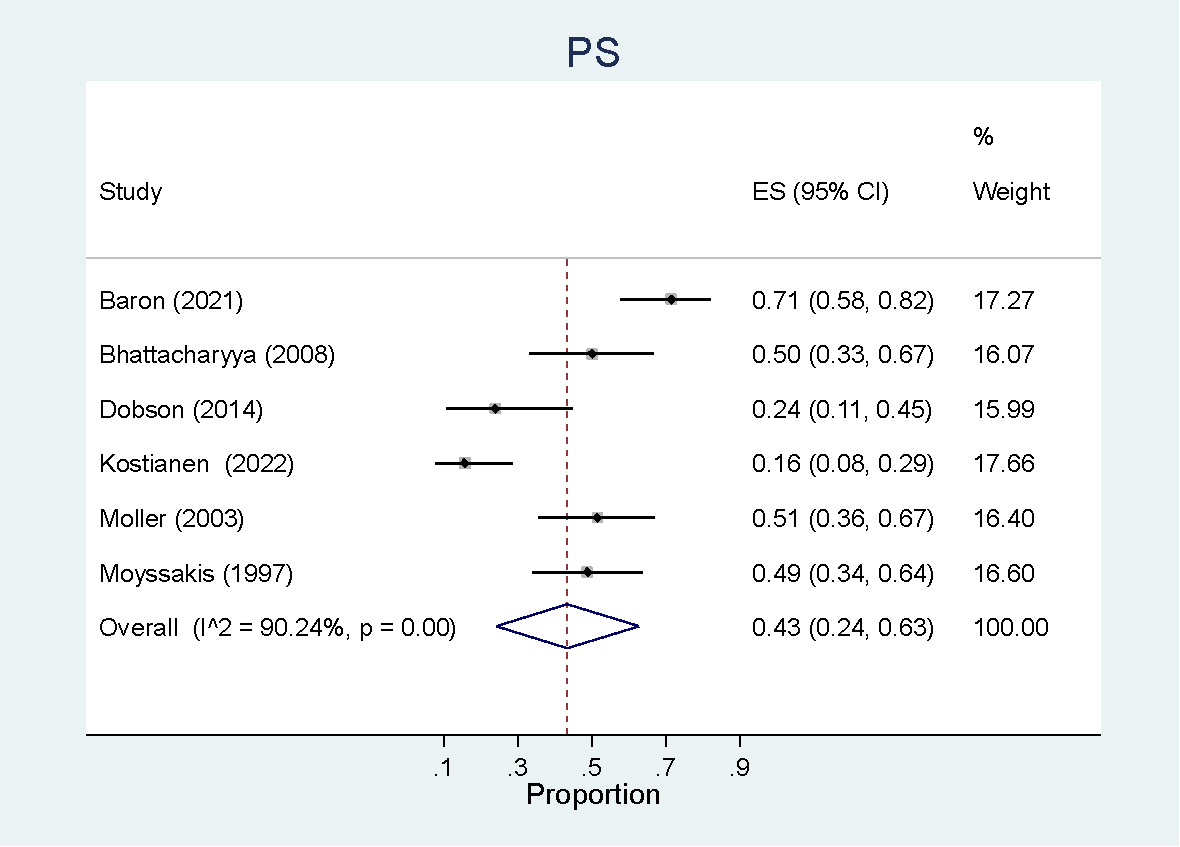


**Supplementary Figure 8. *Echocardiographic finding of pulmonary stenosis (PS) in CS patients***


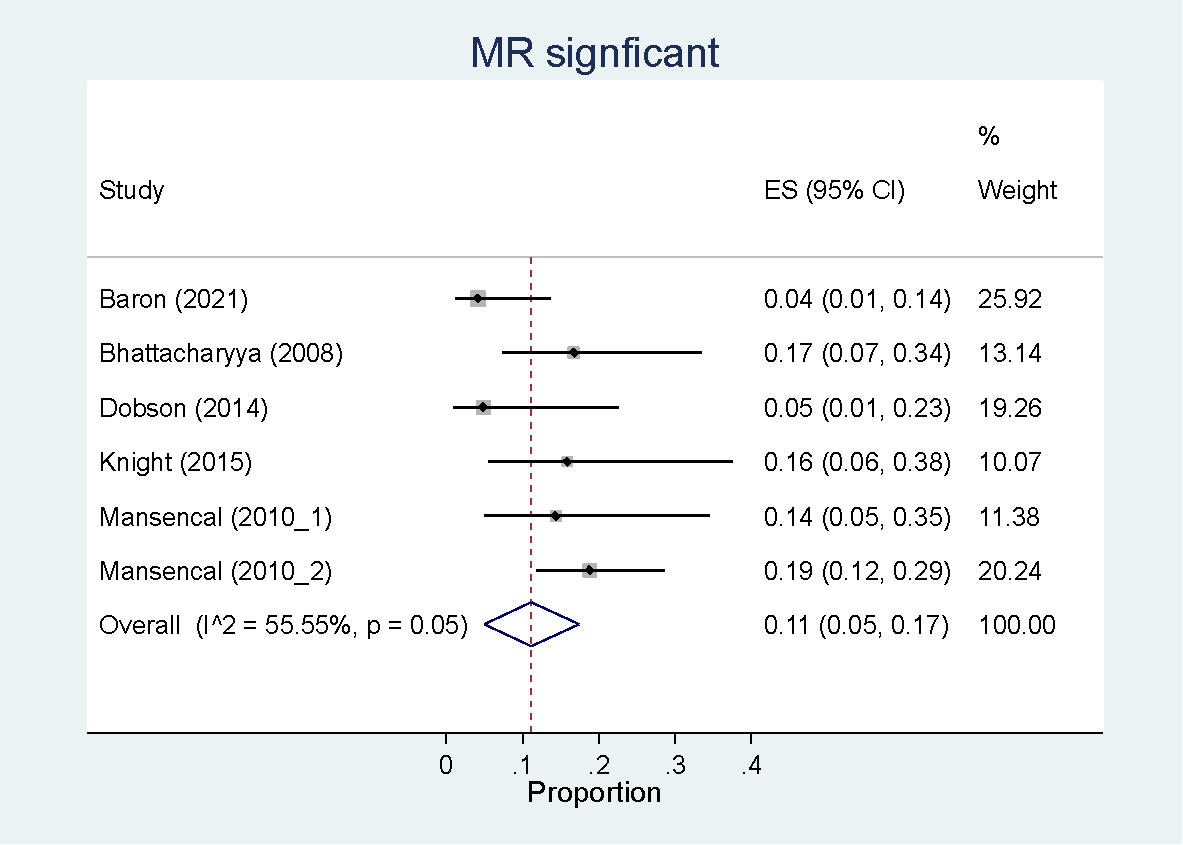


**Supplementary Figure 9. *Echocardiographic finding of significant mitral regurgitation (MR) in CS patients***


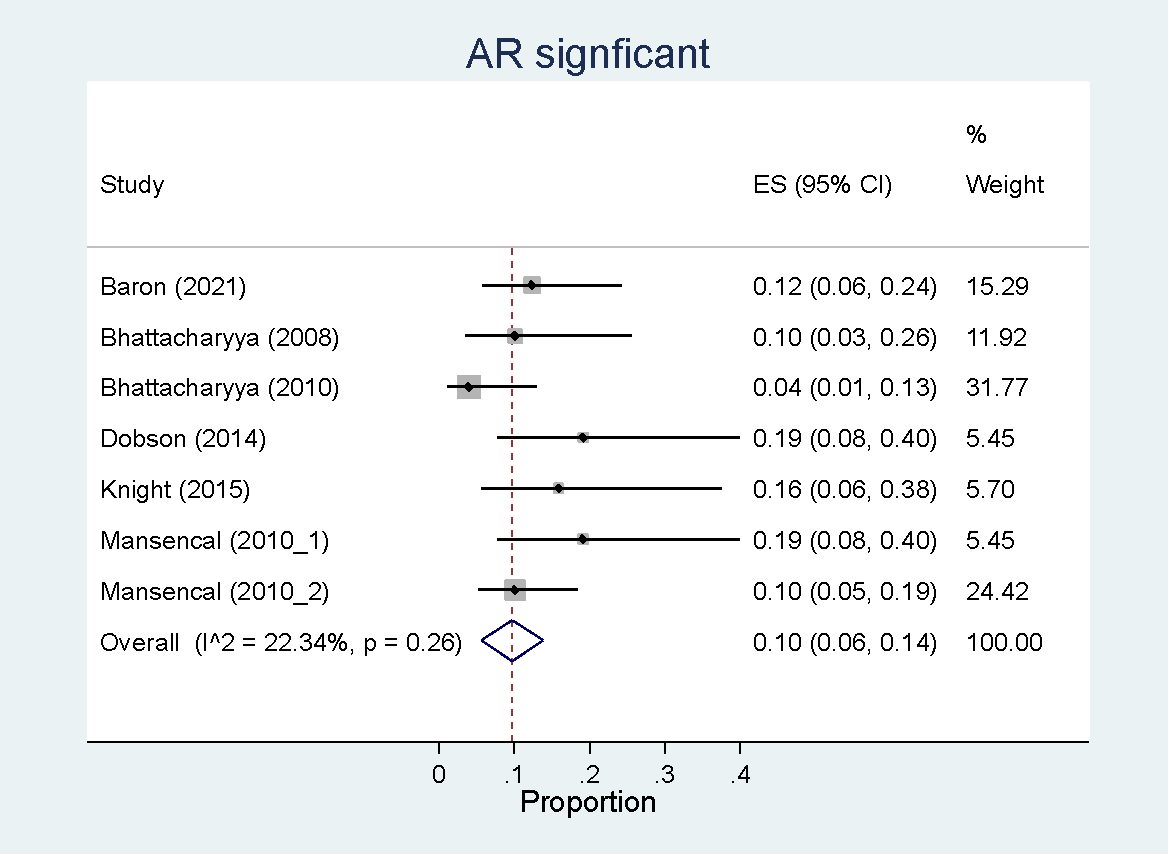


**Supplementary Figure 10. *Echocardiographic finding of significant aortic regurgitation (AR) in CS patients***


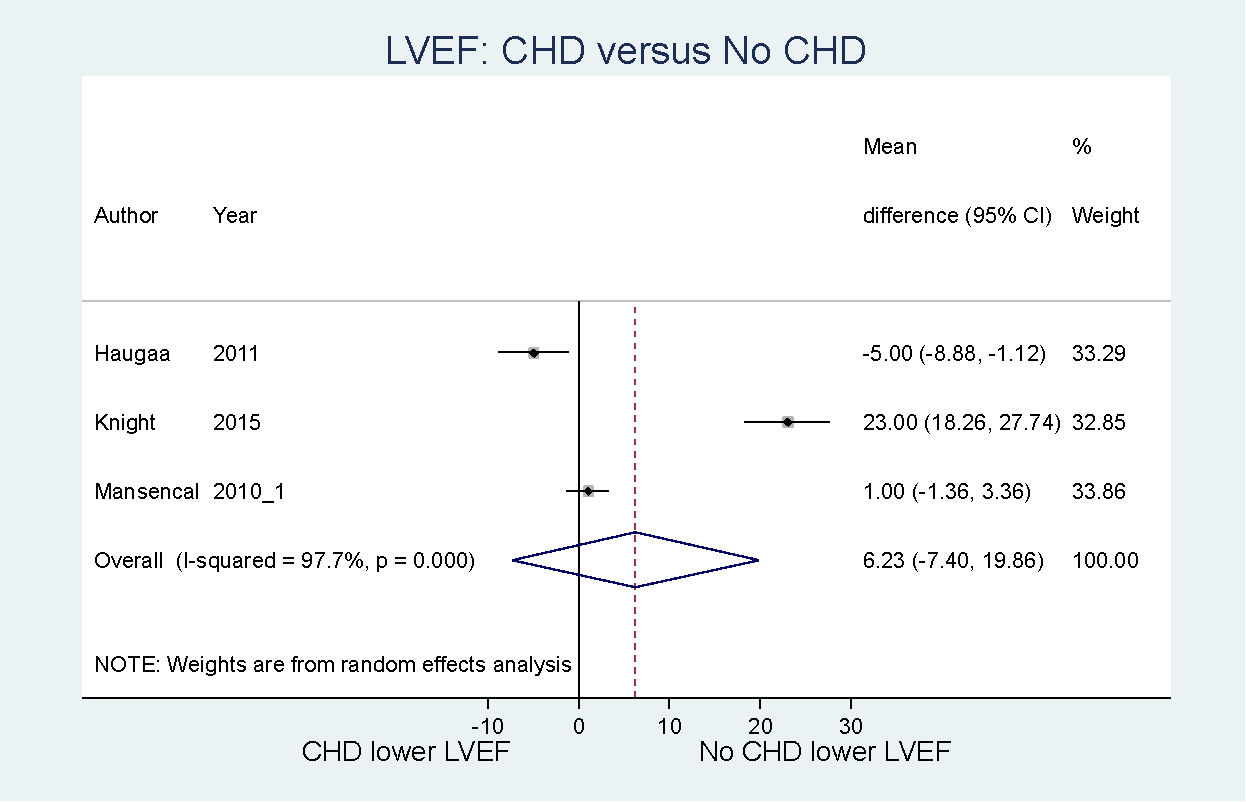


**Supplementary Figure 11: *Echocardiographic finding of left ventricular ejection fraction (LVEF) in CS patients***


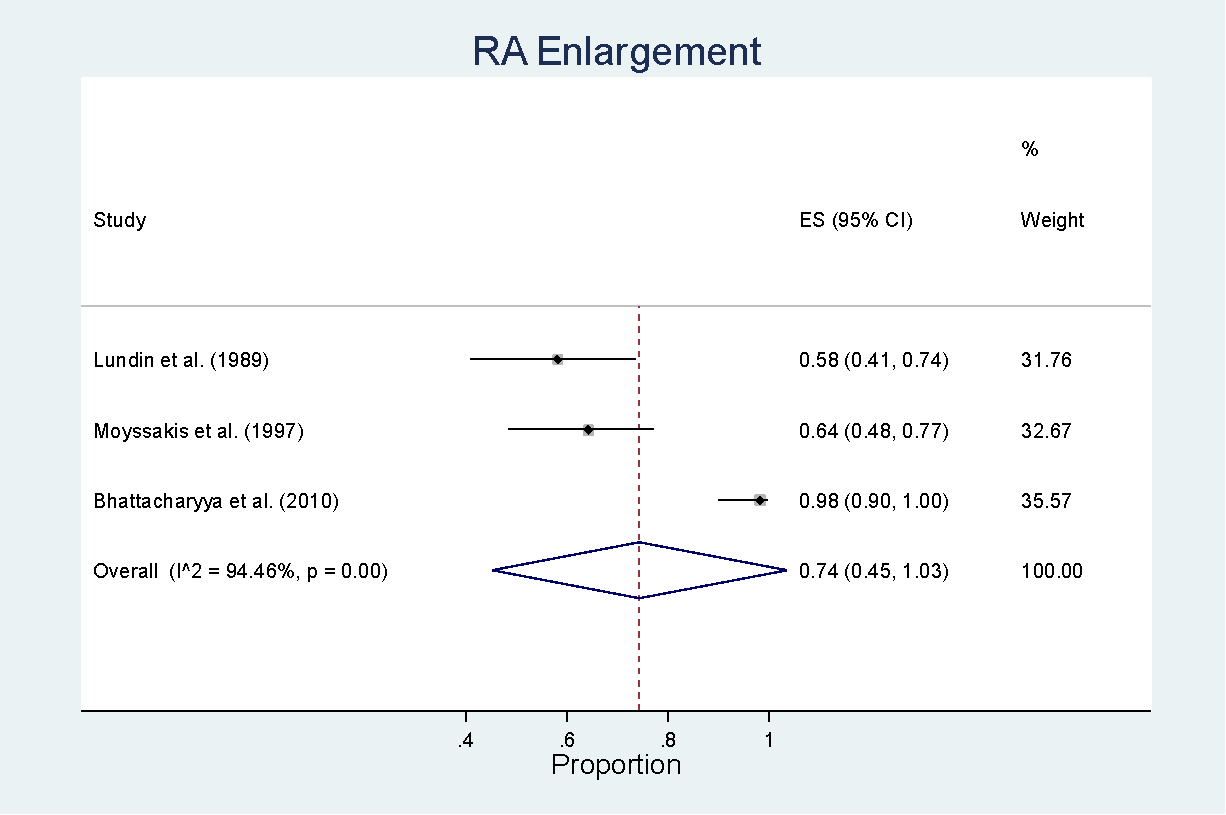


**Supplementary Figure 12: *Echocardiographic finding of right atrial (RA) enlargement in CS patients***


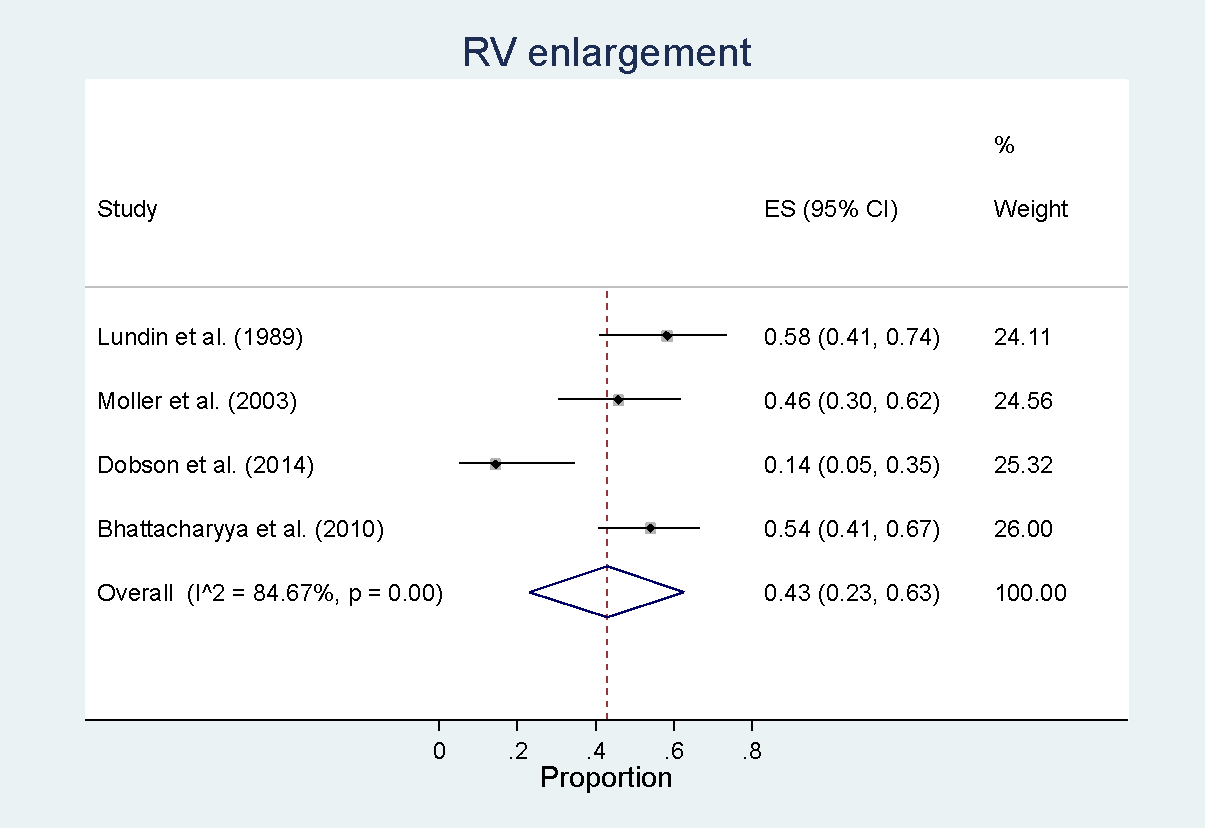


**Supplementary Figure 13: *Echocardiographic finding of right ventricular (RV) enlargement in CS patients***

**
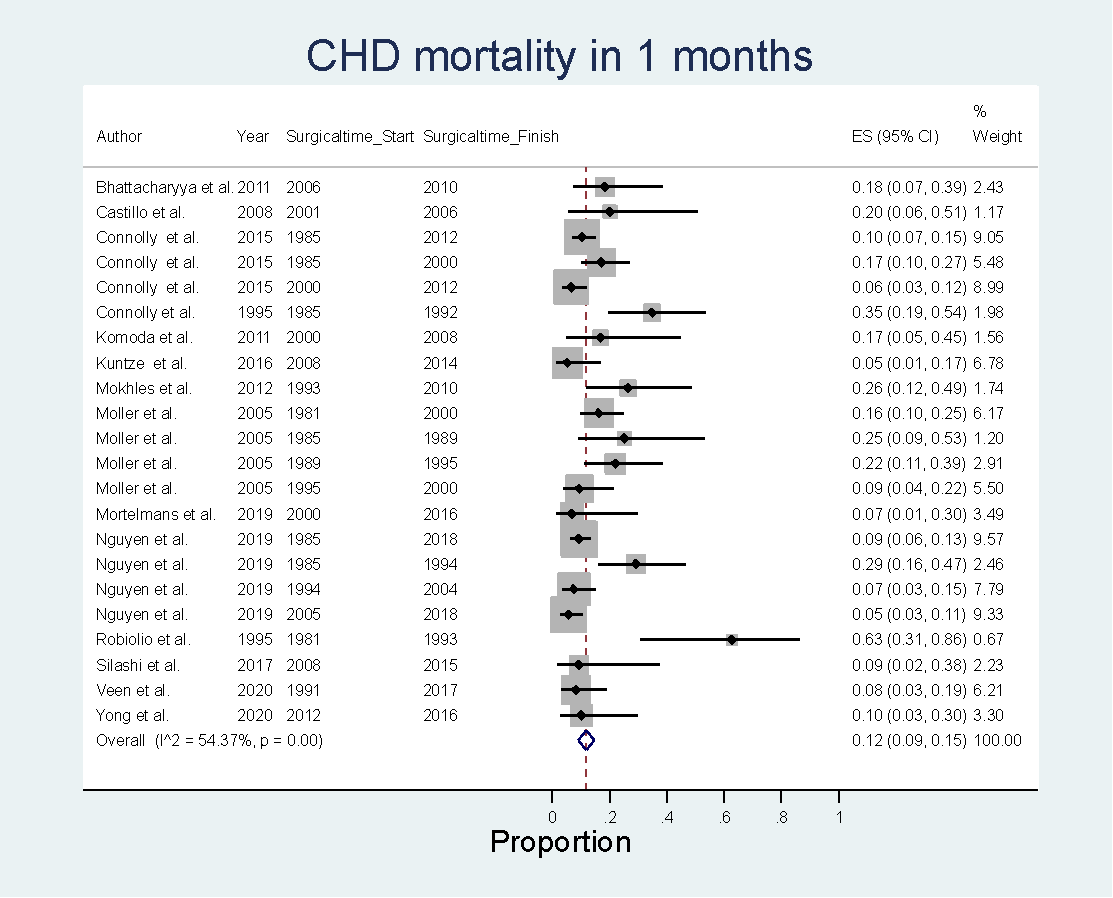
(A)**


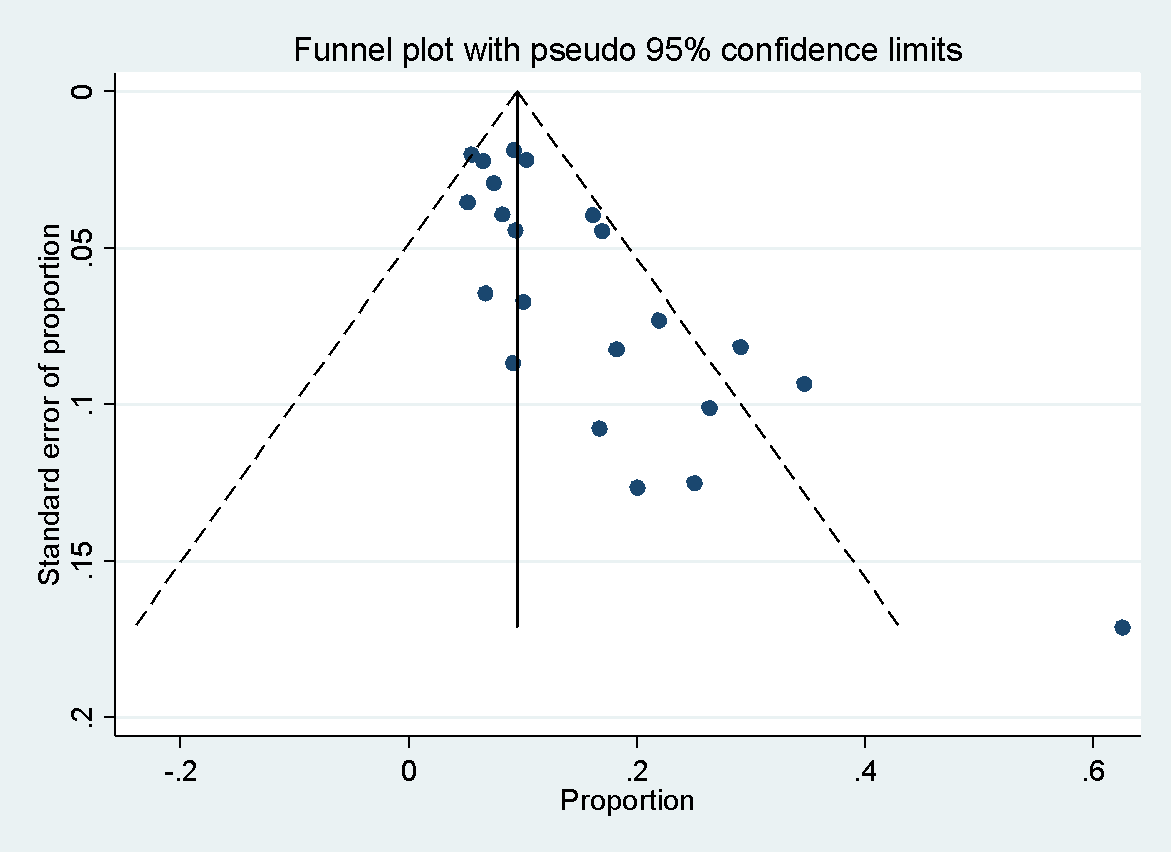
**(B)**

***Supplementary Figure 14: Surgical management outcomes of CHD at 1 month (A) Meta Analysis (B) Funnel Plot***


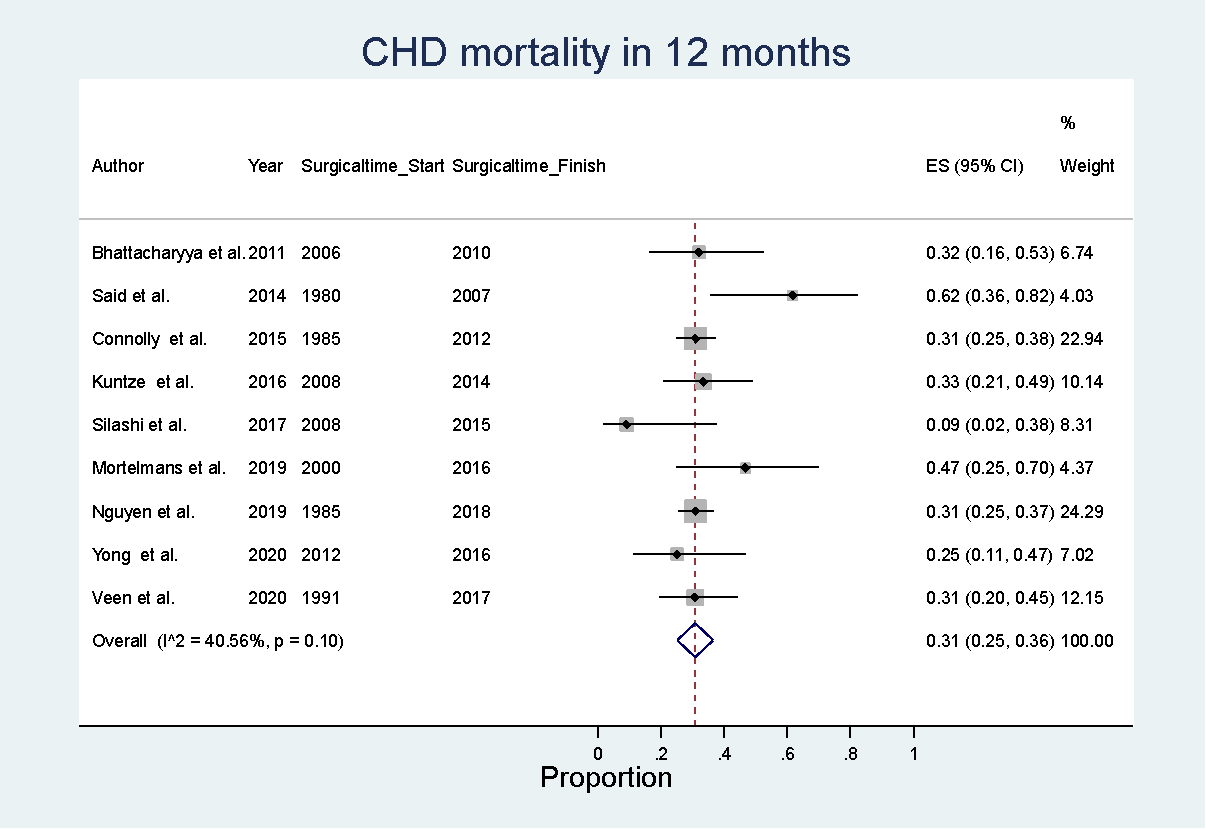


**Supplementary Figure 15: *Surgical management outcomes of CHD at 12 month*s**


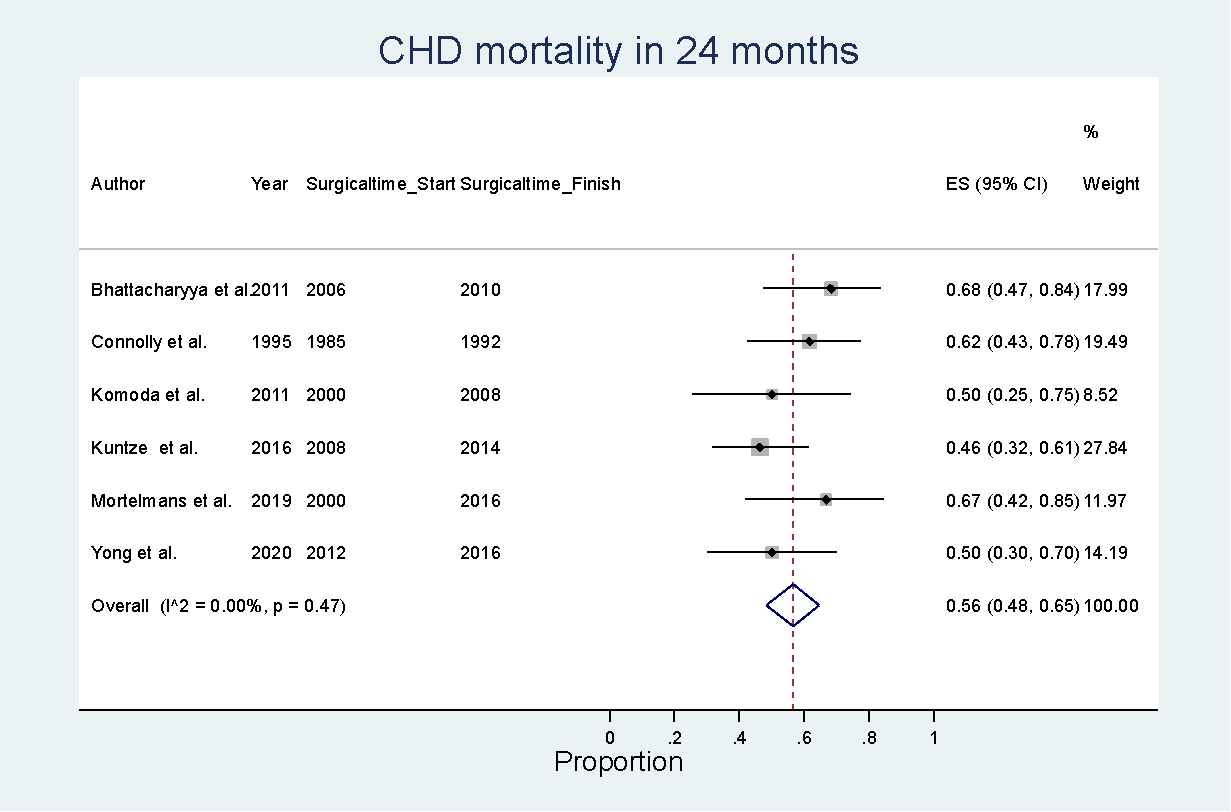


**Supplementary Figure 16:** ***Surgical management outcomes of CHD at 24 months***


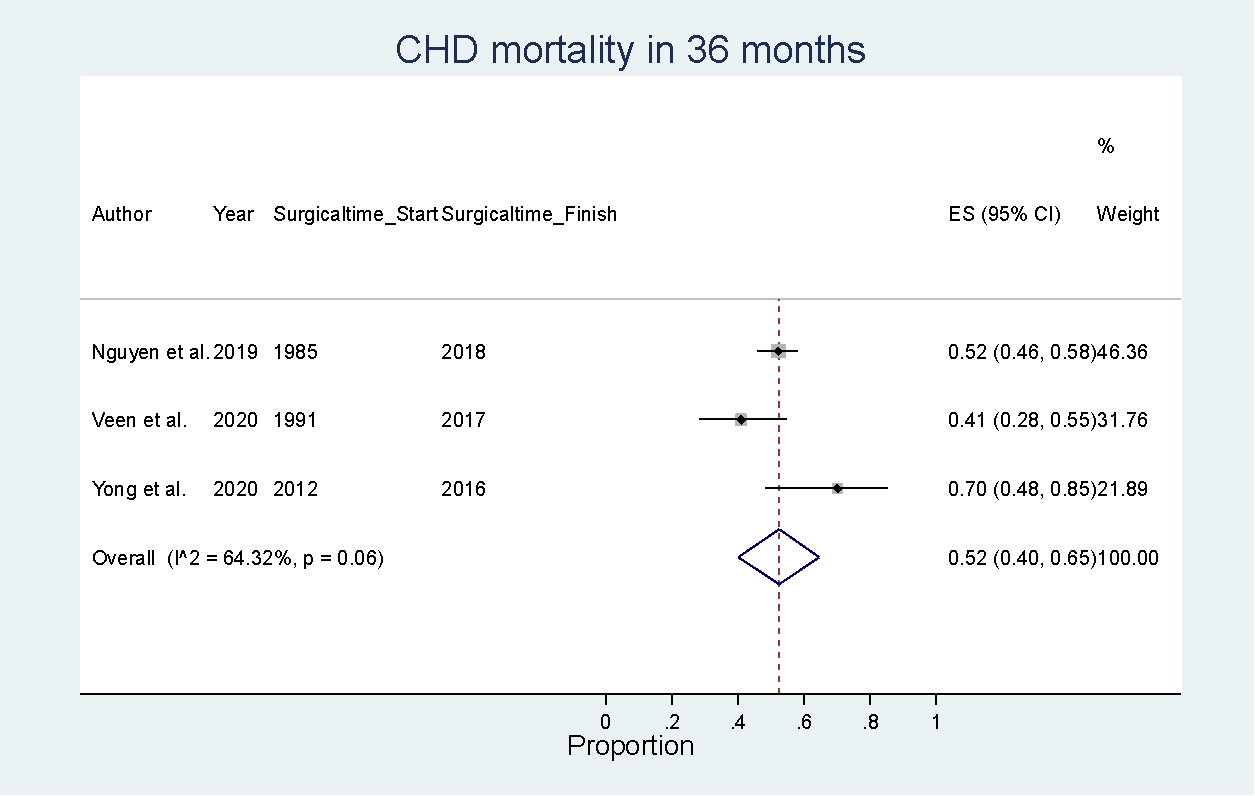


**Supplementary Figure 17: *Surgical management outcomes of CHD at 36 months***


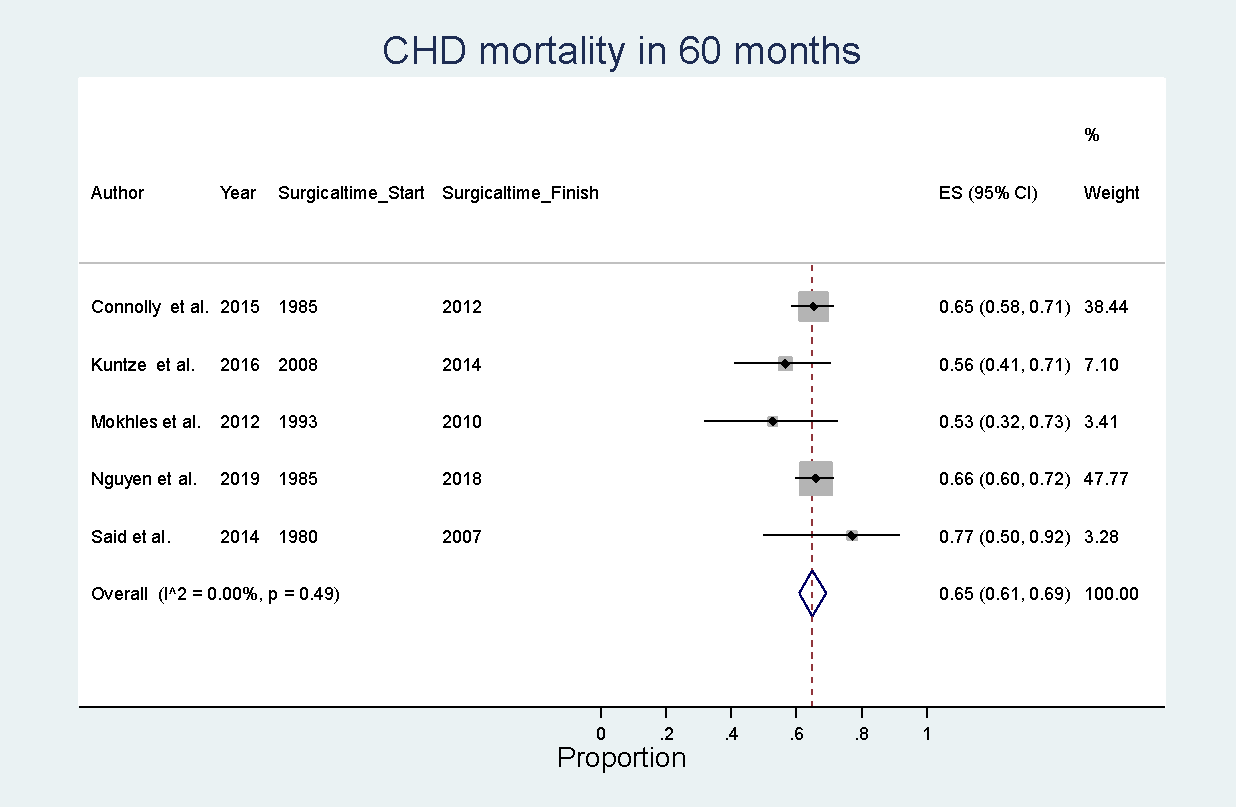


**Supplementary Figure 18: *Surgical management outcomes of CHD at 60 months***

| **Supplementary Table 1: Quality Assessment Tool for Observational Studies - National Heart, Lung and Blood Institute (NHLBI)** | | | | | | | | | | | | | |  |
| --- | --- | --- | --- | --- | --- | --- | --- | --- | --- | --- | --- | --- | --- | --- |
|  |  |  |  |  |  |  |  |  |  |  |  |  |  |  |
| **Study** | **Q1** | **Q2** | **Q3** | **Q4** | **Q5** | **Q6** | **Q7** | **Q8** | **Q9** | **Q10** | **Q11** | **Q12** | **TOTAL** | **Ranking** |
| Bhattacharyya *et al.* 2008a | ✔ | ✔ | NR | ✔ | ✔ | ✔ | NA | ✔ | ✔ | ✔ | NA | NR | 8 | fair |
| Bhattacharyya *et al.* 2008b | ✔ | ✔ | NR | ✔ | ✔ | ✔ | NA | ✔ | ✔ | ✔ | NA | NR | 8 | fair |
| Bhattacharyya *et al.* 2010 | ✔ | ✔ | NR | ✔ | ✔ | ✔ | NA | ✔ | ✔ | ✔ | NA | NR | 8 | fair |
| Bhattacharyya *et al.* 2011 | ✔ | ✔ | NR | ✔ | ✔ | ✔ | NA | ✔ | ✔ | ✔ | NA | NR | 8 | fair |
| Castillo *et al.* 2008 | ✔ | ✔ | NR | NA | ✔ | NA | NA | ✔ | ✔ | ✔ | NA | NR | 6 | fair |
| Connolly et al. 1995 | ✔ | ✔ | NR | NA | ✔ | NA | NA | ✔ | ✔ | ✔ | NA | ✔ | 7 | fair |
| Connolly et al. 2015 | ✔ | ✔ | NR | NA | ✔ | NA | NA | ✔ | ✔ | ✔ | NA | ✔ | 7 | fair |
| Denney *et al.* 1998 | ✔ | ✔ | NR | ✔ | ✔ | ✔ | NA | ✔ | ✔ | ✔ | NA | NR | 8 | fair |
| Dobson *et al.* 2013a | ✔ | ✔ | NR | ✔ | ✔ | ✔ | NA | ✔ | ✔ | ✔ | NA | NR | 8 | fair |
| Dobson *et al.* 2013b | ✔ | ✔ | ✔ | ✔ | ✔ | ✔ | NA | ✔ | ✔ | ✔ | NA | ✔ | 10 | good |
| Haugaa *et al.* 2011 | ✔ | ✔ | NR | ✔ | ✔ | ✔ | NA | ✔ | ✔ | ✔ | NA | NR | 8 | fair |
| Himelman *et al.* 1989 | ✔ | ✔ | NR | ✔ | ✔ | ✔ | NA | ✔ | ✔ | ✔ | NA | NR | 8 | fair |
| Komoda *et al.* 2011 | ✔ | ✔ | NR | NA | ✔ | NA | NA | NA | ✔ | ✔ | NA | ✔ | 6 | fair |
| Kuntze *et al.* 2016 | ✔ | NR | NR | NA | ✔ | NA | NA | NA | ✔ | ✔ | NA | NR | 4 | poor |
| Lundin *et al.* 1989 | ✔ | ✔ | NR | ✔ | ✔ | ✔ | NA | ✔ | ✔ | ✔ | NA | NR | 8 | fair |
| Lundin *et al.* 1990 | ✔ | ✔ | NR | ✔ | ✔ | ✔ | NA | ✔ | ✔ | ✔ | NA | NR | 8 | fair |
| Mansencal et al. 2010a | ✔ | ✔ | NR | ✔ | ✔ | ✔ | NA | ✔ | ✔ | ✔ | NA | ✔ | 9 | fair |
| Mansencal et al. 2010b | ✔ | ✔ | NR | ✔ | ✔ | ✔ | NA | ✔ | ✔ | ✔ | NA | NR | 8 | fair |
| Mokhles et al. 2012 | ✔ | NR | NR | NA | ✔ | NA | NA | NA | ✔ | ✔ | NA | NR | 4 | fair |
| Moller et al. 2005 | ✔ | ✔ | NR | ✔ | ✔ | ✔ | NA | ✔ | ✔ | ✔ | NA | NR | 8 | good |
| Moller et al. 2021 | ✔ | ✔ | NR | ✔ | ✔ | ✔ | NA | ✔ | ✔ | ✔ | NA | ✔ | 9 | good |
| Mortelmans et al. 2019 | ✔ | NR | NR | NA | ✔ | NA | NA | NA | ✔ | ✔ | NA | NR | 4 | poor |
| Moyssakis et al. 1997 | ✔ | ✔ | NR | ✔ | NR | NR | NA | NR | ✔ | ✔ | NA | NR | 5 | poor |
| Nguyen et al. 2019 | ✔ | ✔ | NR | ✔ | ✔ | ✔ | NA | ✔ | ✔ | ✔ | NA | NR | 8 | fair |
| Robiolio et al. 1995 | ✔ | ✔ | NR | ✔ | ✔ | ✔ | NA | ✔ | ✔ | ✔ | NA | NR | 8 | fair |
| Said et al. 2014 | ✔ | ✔ | NR | NA | ✔ | NA | NA | NA | ✔ | ✔ | NA | NR | 5 | poor |
| Silaschi et al. 2017 | ✔ | ✔ | NR | NA | ✔ | NA | NA | NA | ✔ | ✔ | NA | NR | 5 | poor |
| Veen et al. 2020 | ✔ | ✔ | NR | NA | ✔ | NA | NA | NA | ✔ | ✔ | NA | NR | 5 | poor |
| Yong et al. 2020 | ✔ | ✔ | NR | NA | ✔ | NA | NA | NA | ✔ | ✔ | NA | NR | 5 | poor |
| Zuetenhorst *et al.* 2004 | ✔ | ✔ | NR | ✔ | ✔ | ✔ | NA | ✔ | ✔ | ✔ | NA | NR | 8 | fair |
