# Supplementary material for: A systematic review and meta-analysis of the diagnosis and surgical management of carcinoid heart disease
Source: Front Cardiovasc Med. 2024 Mar 20;11:1353612. doi: 10.3389/fcvm.2024.1353612 (PMC10987853; doi:10.3389/fcvm.2024.1353612)
Supplement: Supplementary file 2 [file Table2.docx]

**Appendix 1:** PECO:

Population: Patients with carcinoid syndrome
Exposure: Carcinoid Heart Disease
Outcome: Biomarkers, surgical outcomes, echocardiographic findings.

**Appendix 2:** Search strategy

**Ovid MEDLINE(R) and Epub Ahead of Print, In-Process, In-Data-Review & Other Non-Indexed Citations and Daily <1946 to March 12, 2021>**

1 Carcinoid Heart Disease/ 492

2 (carcinoid adj3 (heart or HD or cardi*)).ti,ab,kf. 628

3 Hedinger* syndrome.ti,ab,kf. 15

4 1 or 2 or 3 753

5 4 not (Animals/ not (Animals/ and Humans/)) 747

**Embase <1974 to 2021 March 12>**

1 (carcinoid adj3 (heart or HD or cardi*)).ti,ab,kw. 862

2 Hedinger* syndrome.ti,ab,kw. 18

3 1 or 2 864

4 limit 3 to conference abstract 219

5 3 not 4 645

6 5 not ((exp animal/ or nonhuman/) not exp human/) 638

**EBM Reviews - Cochrane Central Register of Controlled Trials <February 2021>**

1 Carcinoid Heart Disease/ 2

2 (carcinoid adj3 (heart or HD or cardi*)).ti,ab,kw. 15

3 Hedinger* syndrome.ti,ab,kw. 0

4 1 or 2 or 3 15

**Google Scholar**

"carcinoid heart disease"|"hedinger syndrome"

4,430 results - first 200 exported

**ClinicalTrials.gov**

"Carcinoid Heart Disease" OR "Hedinger syndrome"

3 results

**Appendix 3:** Reasons for excluding studies (3 examples).

| **Study** | **Reason for Exclusion** |
| --- | --- |
| Nitu et al. 2022 | There was no explicit mention of patients with Carcinoid Heart Disease (CHD) |
| Peczkowska et al. 2022 | This was a systematic review of the literature. |
| Tanaka 2020 | Did not report on biomarkers, surgical outcomes or echocardiographic markers of interest. |
